# Supplementary figures and images for: Sexist textbooks: Automated analysis of gender bias in 1,255 books from 34 countries
Source: PLoS One. 2024 Oct 9;19(10):e0310366. doi: 10.1371/journal.pone.0310366 (PMC11463758; doi:10.1371/journal.pone.0310366)

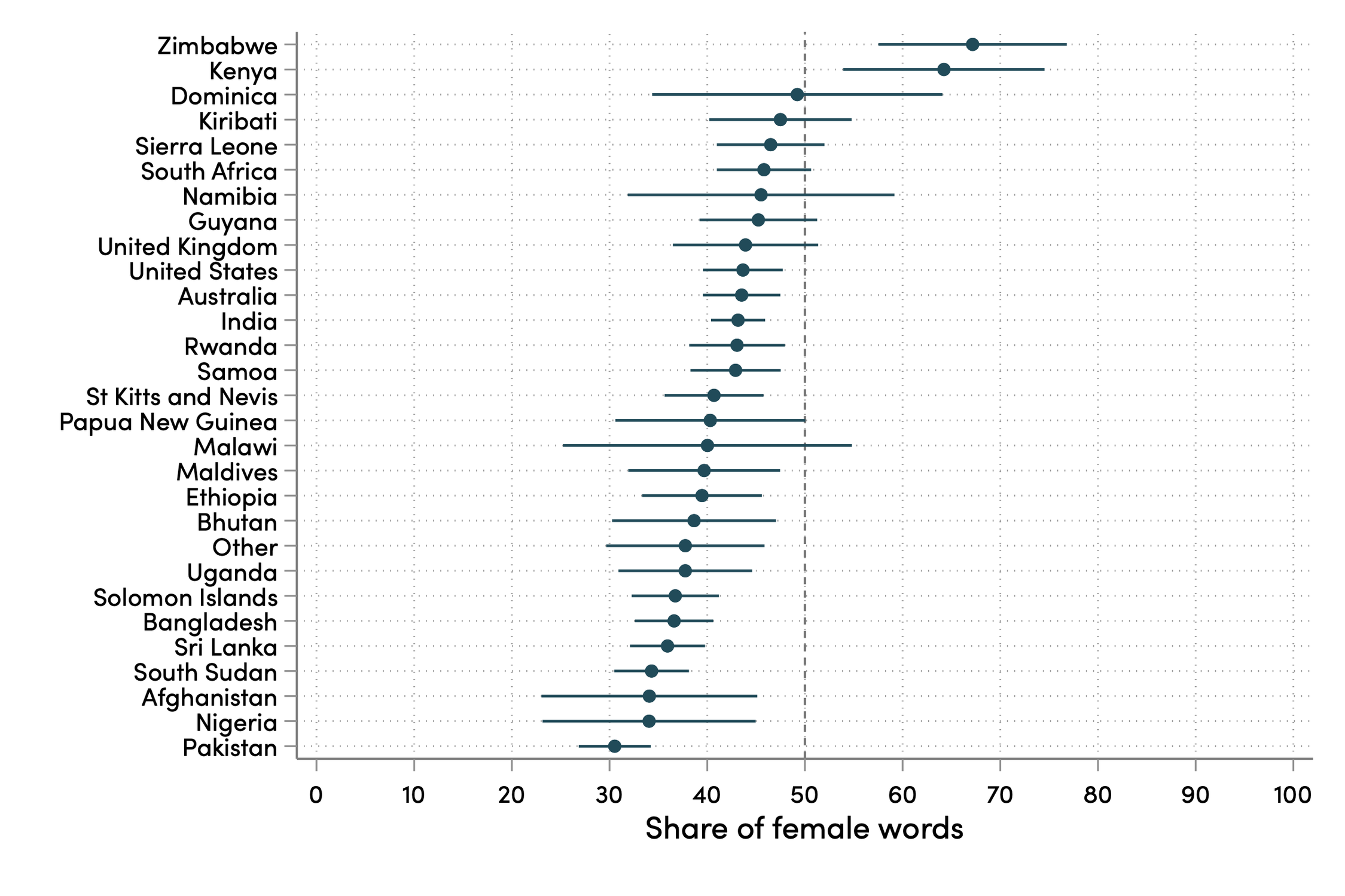

Supplement: S1 Fig — Note: These figures show the predicted mean share of gendered words that are female. Here, word counts for each gender include names identified as male or female. This measure is first calculated for each individual book, which are then estimated as a function of country, subject, grade, and (log) book length. We exclude countries with fewer than five books in our corpus. The high-income countries are Australia, the United Kingdom, and the United States. The low and middle income countries with donor-funded books are Bhutan, Ethiopia, Guyana, Kenya, Lesotho, Liberia, Namibia, Nigeria, Papua New Guinea, Samoa, Sierra Leone, South Africa, South Sudan, Tonga, Zambia, and Zimbabwe. (ZIP) [file pone.0310366.s001.zip › S1 Fig (A).tif]

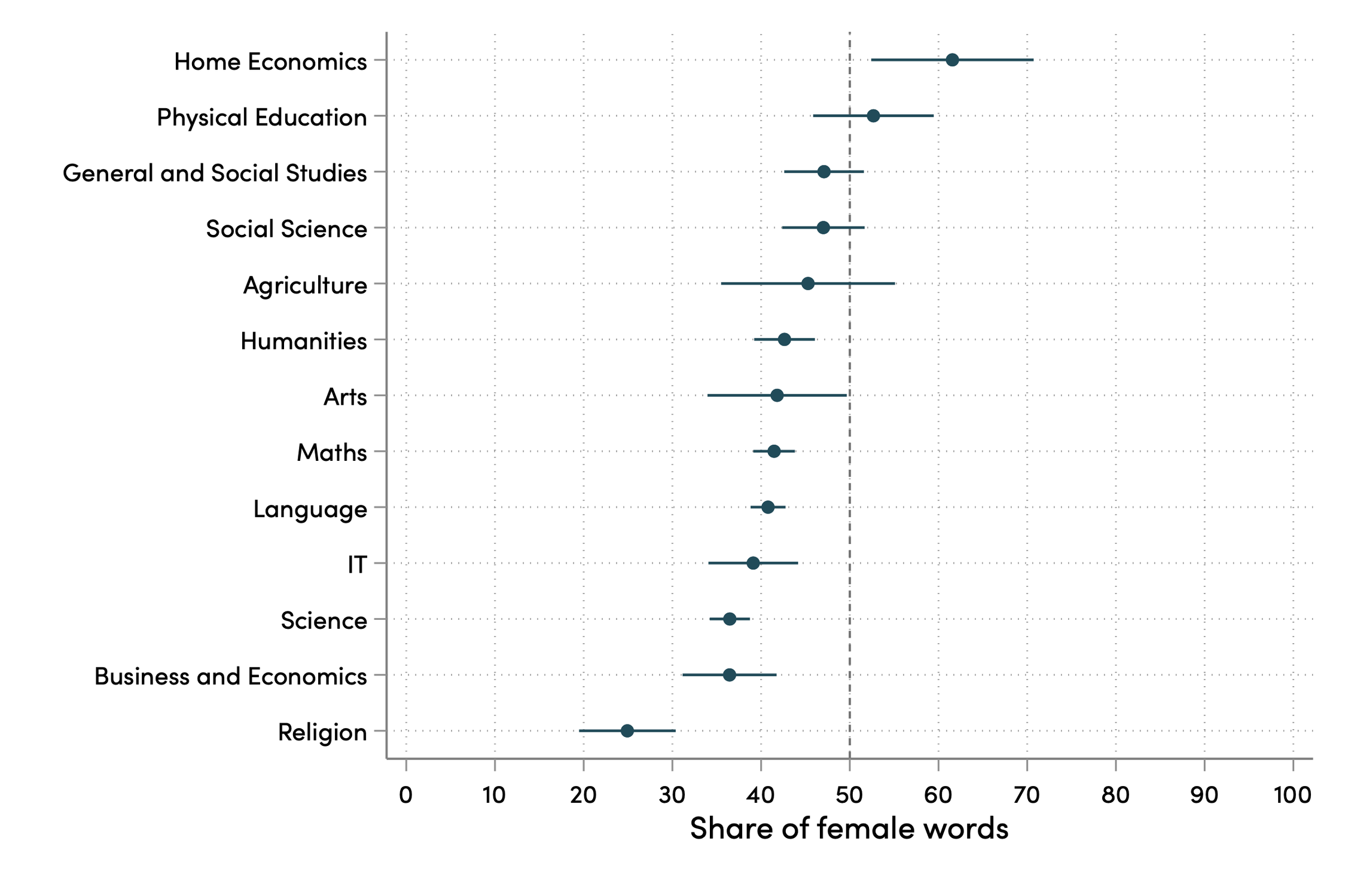

Supplement: S1 Fig — Note: These figures show the predicted mean share of gendered words that are female. Here, word counts for each gender include names identified as male or female. This measure is first calculated for each individual book, which are then estimated as a function of country, subject, grade, and (log) book length. We exclude countries with fewer than five books in our corpus. The high-income countries are Australia, the United Kingdom, and the United States. The low and middle income countries with donor-funded books are Bhutan, Ethiopia, Guyana, Kenya, Lesotho, Liberia, Namibia, Nigeria, Papua New Guinea, Samoa, Sierra Leone, South Africa, South Sudan, Tonga, Zambia, and Zimbabwe. (ZIP) [file pone.0310366.s001.zip › S1 Fig (B).tif]

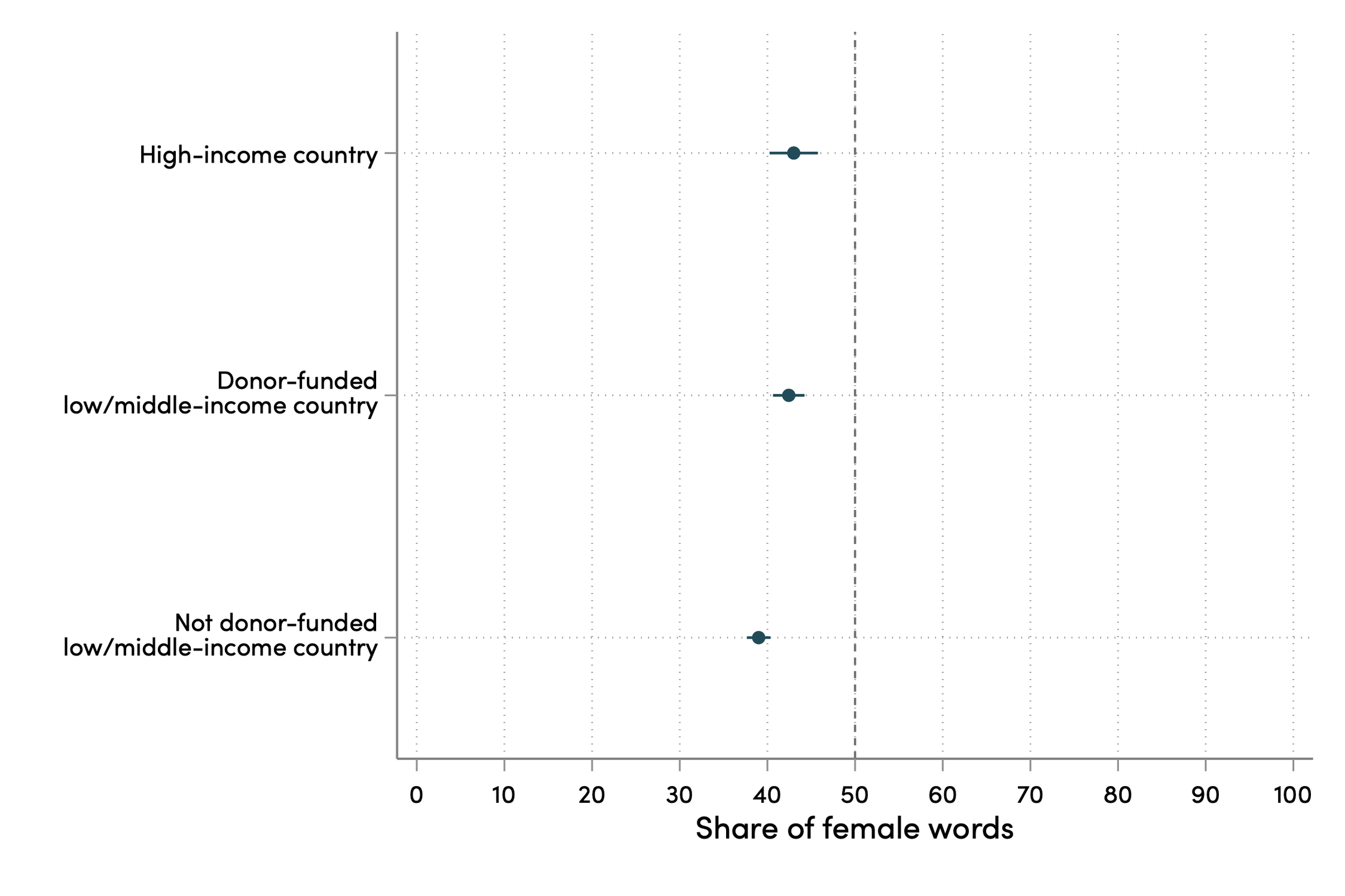

Supplement: S1 Fig — Note: These figures show the predicted mean share of gendered words that are female. Here, word counts for each gender include names identified as male or female. This measure is first calculated for each individual book, which are then estimated as a function of country, subject, grade, and (log) book length. We exclude countries with fewer than five books in our corpus. The high-income countries are Australia, the United Kingdom, and the United States. The low and middle income countries with donor-funded books are Bhutan, Ethiopia, Guyana, Kenya, Lesotho, Liberia, Namibia, Nigeria, Papua New Guinea, Samoa, Sierra Leone, South Africa, South Sudan, Tonga, Zambia, and Zimbabwe. (ZIP) [file pone.0310366.s001.zip › S1 Fig (C).tif]

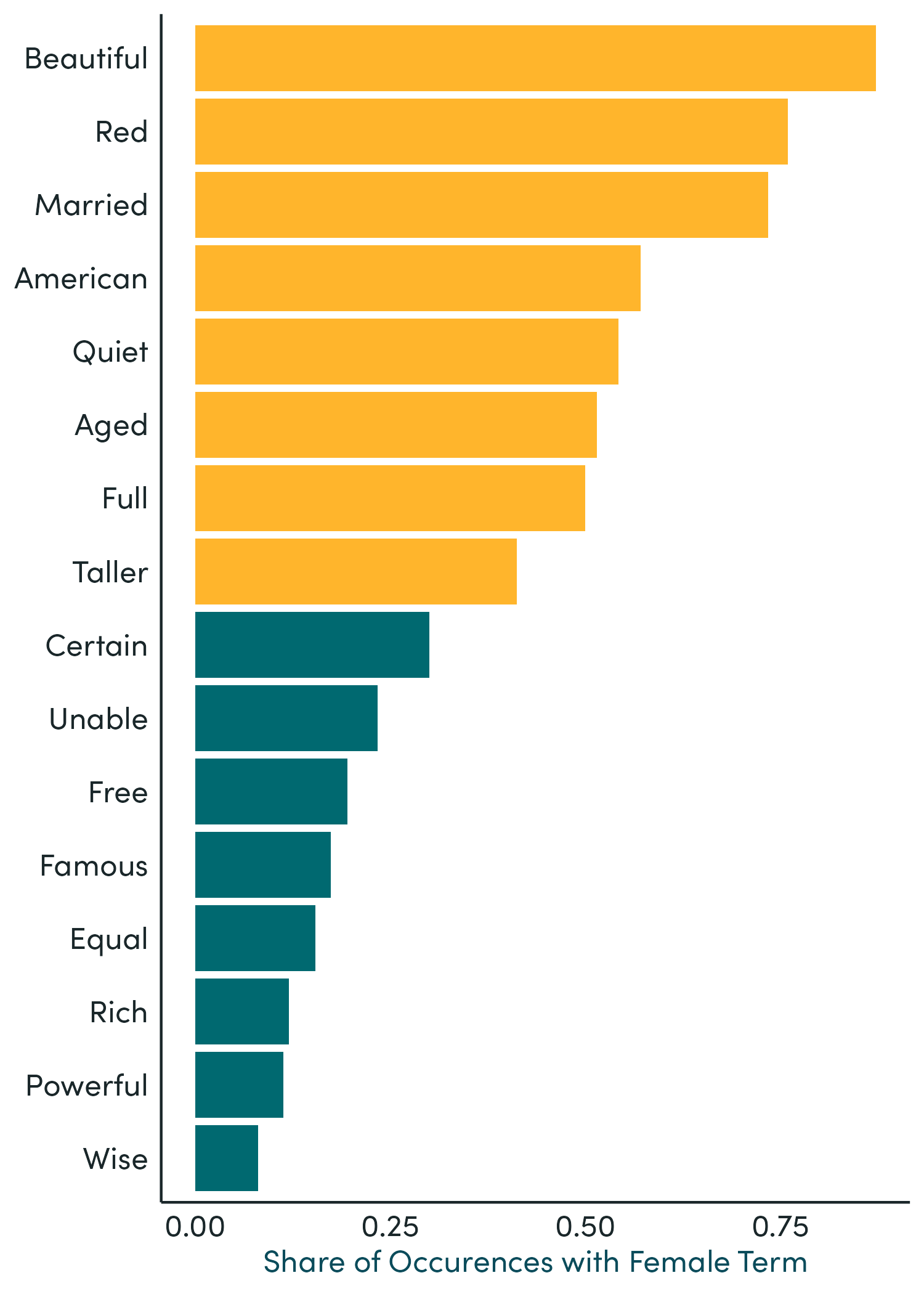

Supplement: S2 Fig — Note: This figure shows the share of occurrences of adjectives used to describe people, which were used for female terms or nouns. We show here the 12 adjectives which were the most skewed toward either gender across UK/US/AUS, South Asia, and Sub-Saharan Africa, provided that they occurred at least 5 times in textbooks for each region. (TIF) [file pone.0310366.s002.tif]

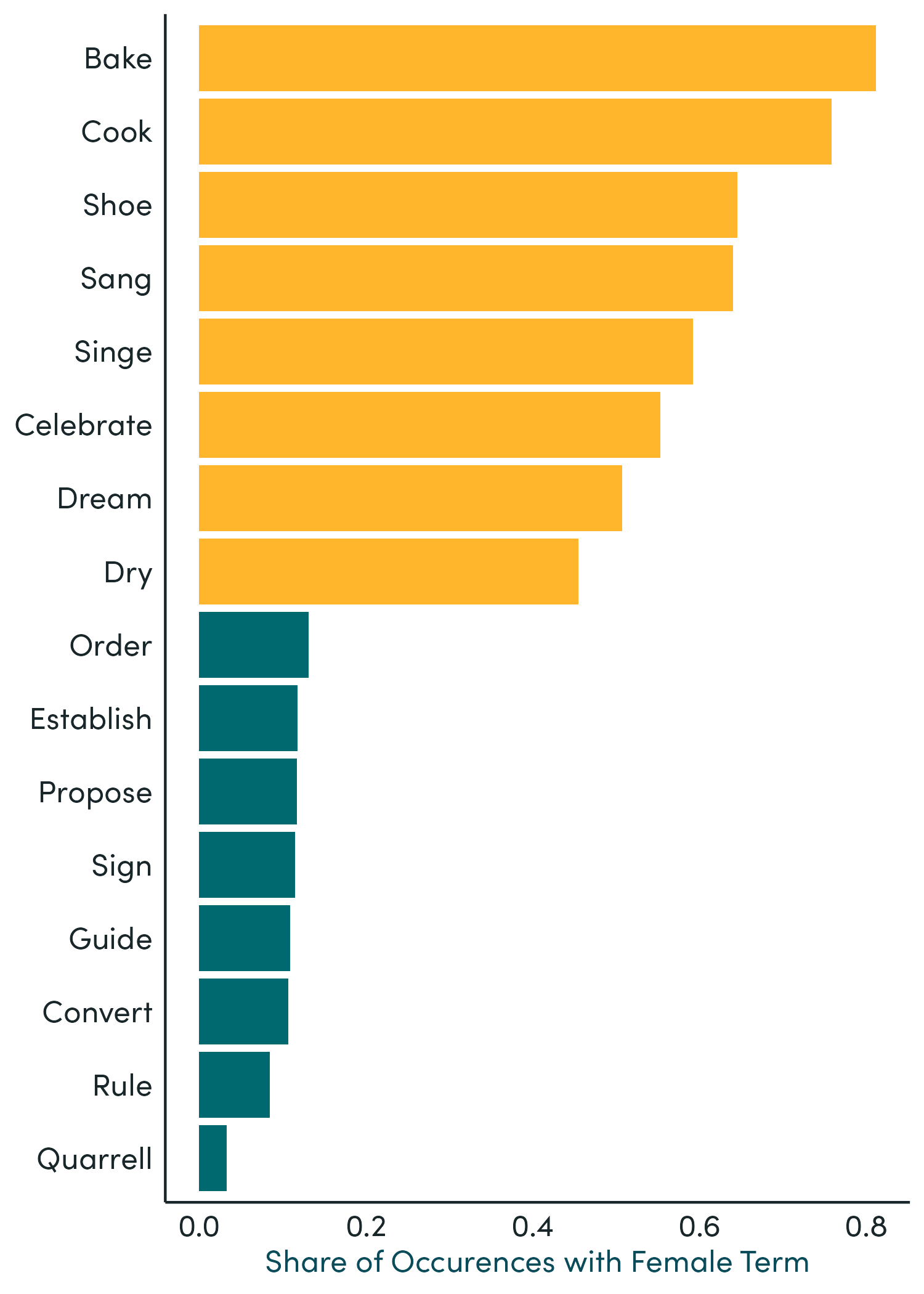

Supplement: S3 Fig — Note: This figure shows the share of occurrences of verbs used to describe people, which were used for female terms or nouns. We show here the 12 verbs—to be precise, the lemmas of verbs—which were the most skewed toward either gender across UK/US/AUS, South Asia, and Sub-Saharan Africa, provided that they occurred at least 5 times in textbooks for each region. (TIF) [file pone.0310366.s003.tif]

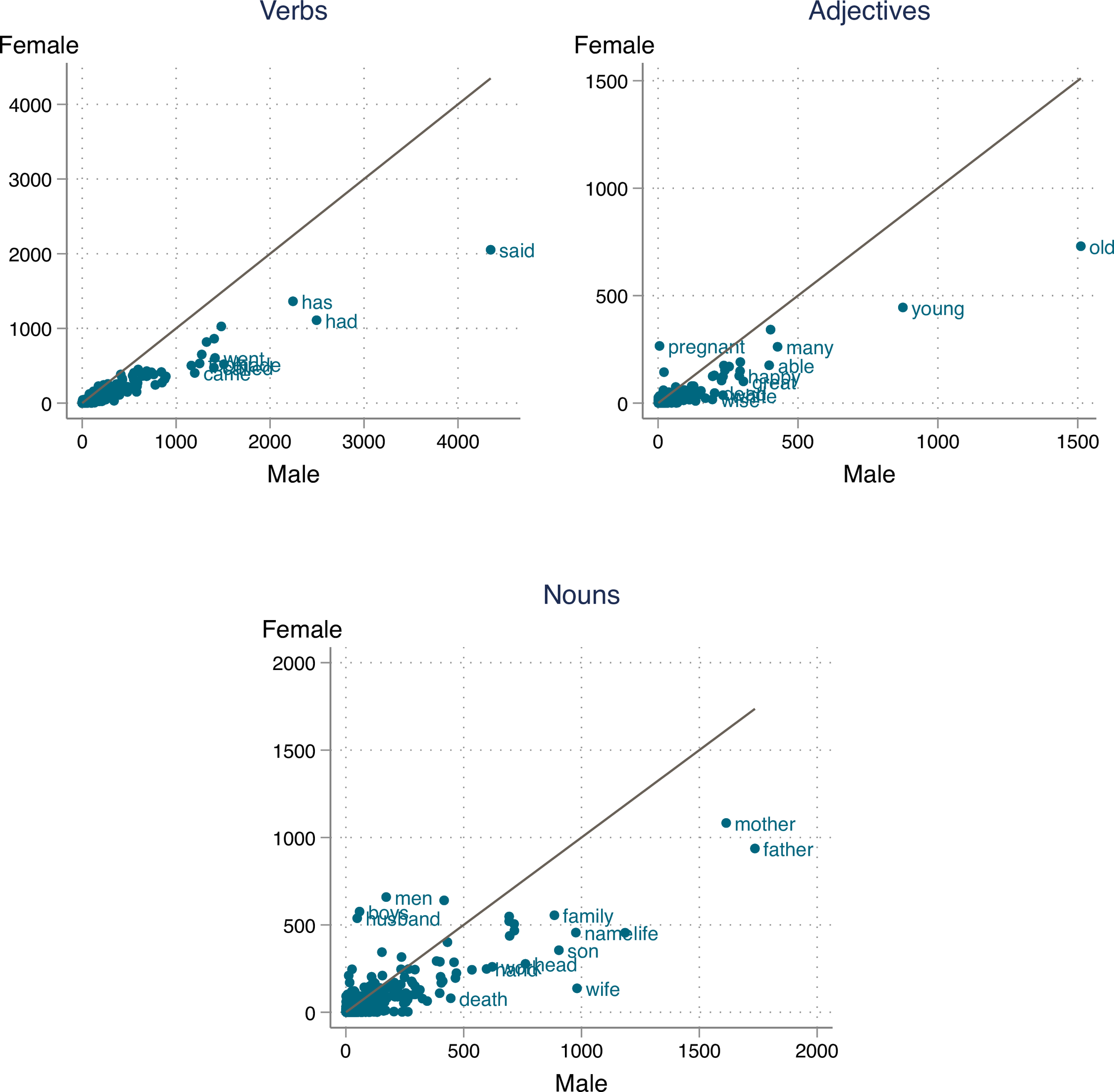

Supplement: S4 Fig — Note: These figures show the counts of the number of occurrences of gendered words alongside different specific adjectives, verbs, and nouns. The gendered words used are those contained in S1 File and the names described in Section. We identify adjectives, verbs, and nouns using the dependency parsing approach described in Section. (TIF) [file pone.0310366.s004.tif]

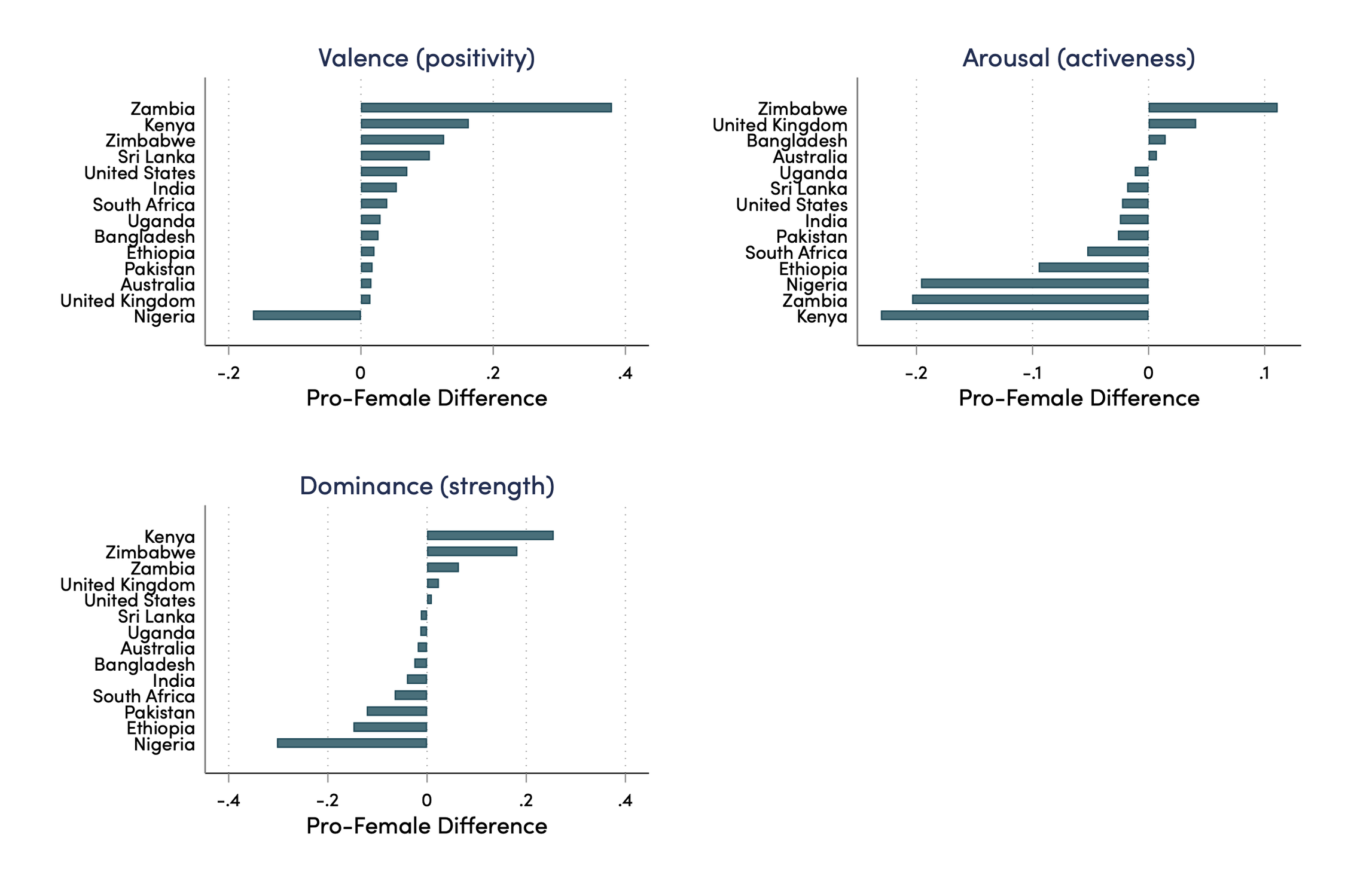

Supplement: S5 Fig — Note: These figures show the differences in average scores (in Valence, Arousal, and Dominance) for adjectives and verbs used for female compared to male gender terms. The difference is shown by country. (TIF) [file pone.0310366.s005.tif]

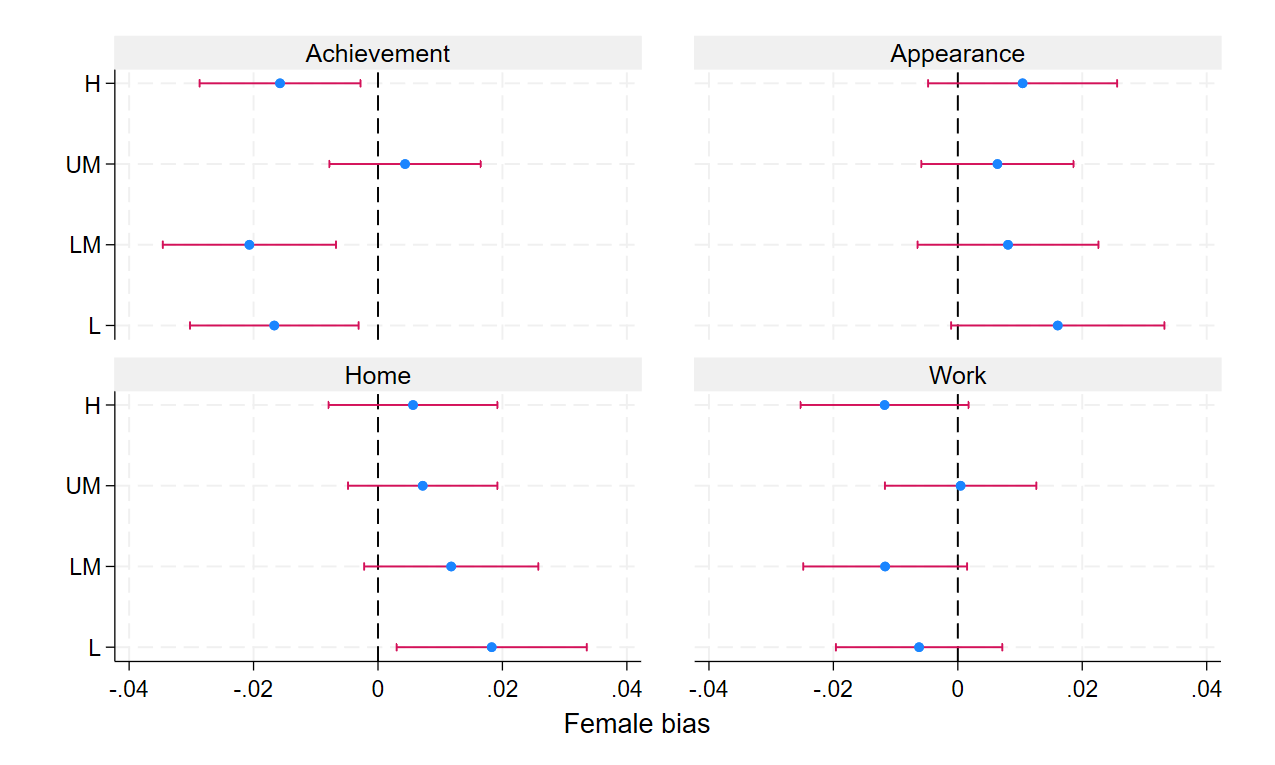

Supplement: S6 Fig — Note: Female bias is calculated as the difference between the average cosine similarity of the theme word with the set of female gender terms and the average similarity of the theme word with the set of male gender terms. Confidence intervals are calculated as the standard deviation for this statistic, over 50 bootstrap samples, where samples are generated by sampling all sentences in our corpus with replacement, until a sample has as many sentences as the original corpus. H indicates high-income countries, which are Australia, Canada, the United Kingdom, the United States, Guyana, and St Kitts and Nevis. UM are upper-middle-income countries which are Belize, Dominica, Jamaica, Namibia, South Africa, and Tonga. LM are lower-middle-income countries, which are Bangladesh, India, Pakistan, Kenya, Nigeria, Zambia, Zimbabwe, Bhutan, Kiribati, Papua New Guinea, Samoa, Solomon Islands, Sri Lanka, and Lesotho. L are low-income countries, which are Afghanistan, Ethiopia, Rwanda, South Sudan, Liberia, Sierra Leone, and Uganda. (TIF) [file pone.0310366.s006.tif]

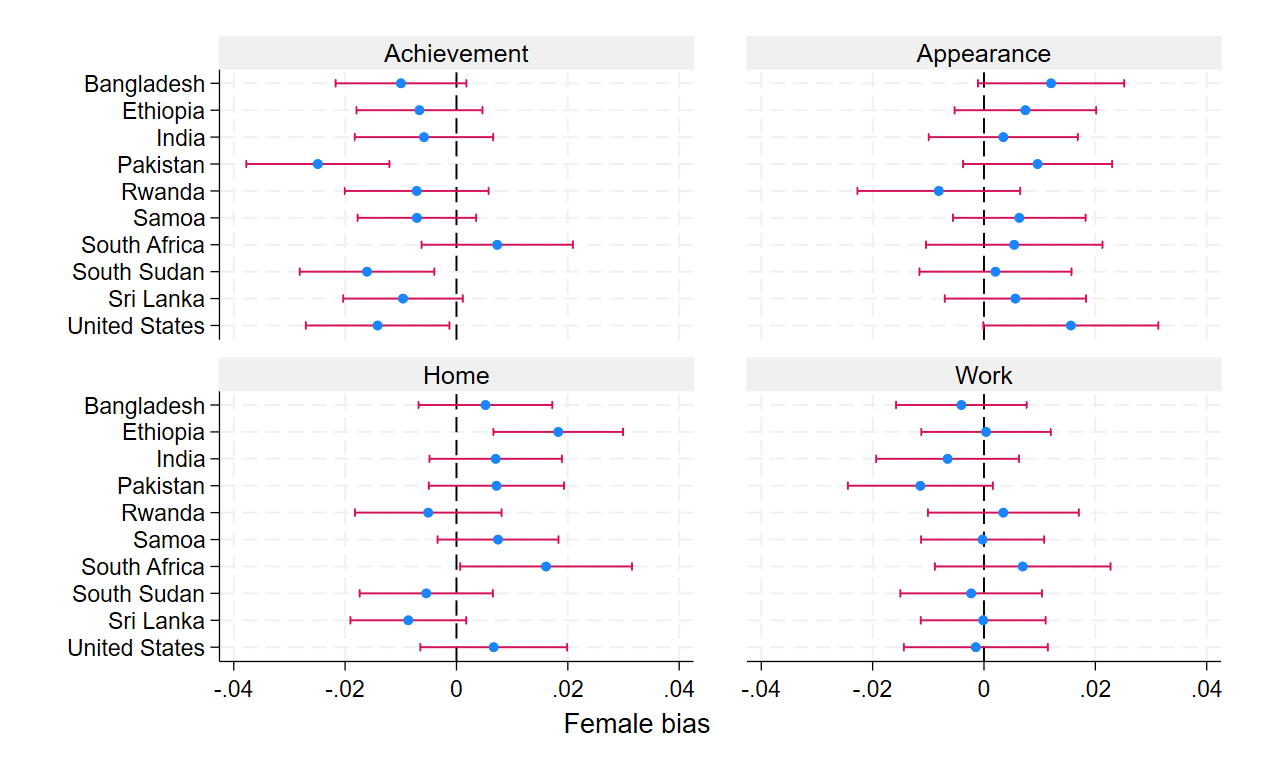

Supplement: S7 Fig — Note: Biases are shown only for the 10 countries in our corpus with at least 1.5 million tokens, as embeddings generated for other countries will be estimated unreliably. Female bias is calculated as the difference between the average cosine similarity of the theme word with the set of female gender terms, and the average similarity of the theme word with the set of male gender terms. Confidence intervals are calculated as the standard deviation for this statistic, over 50 bootstrap samples, where samples are generated by sampling all sentences in our corpus with replacement, until a sample has as many sentences as the original corpus. (TIF) [file pone.0310366.s007.tif]

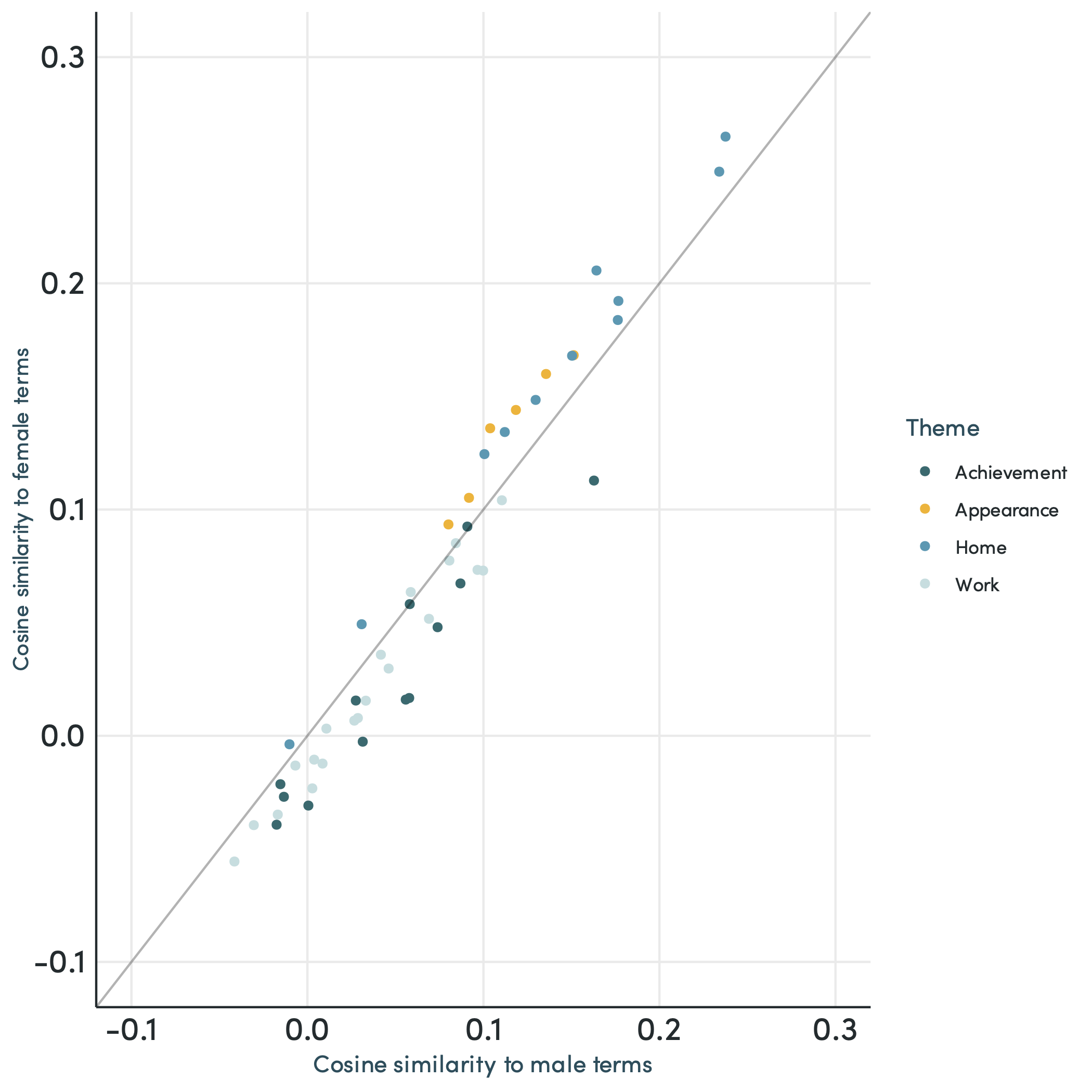

Supplement: S8 Fig — Note: Words above the 45° line are discussed more frequently in relation to women and girls, words below the line more frequently in relation to men and boys. (TIF) [file pone.0310366.s008.tif]

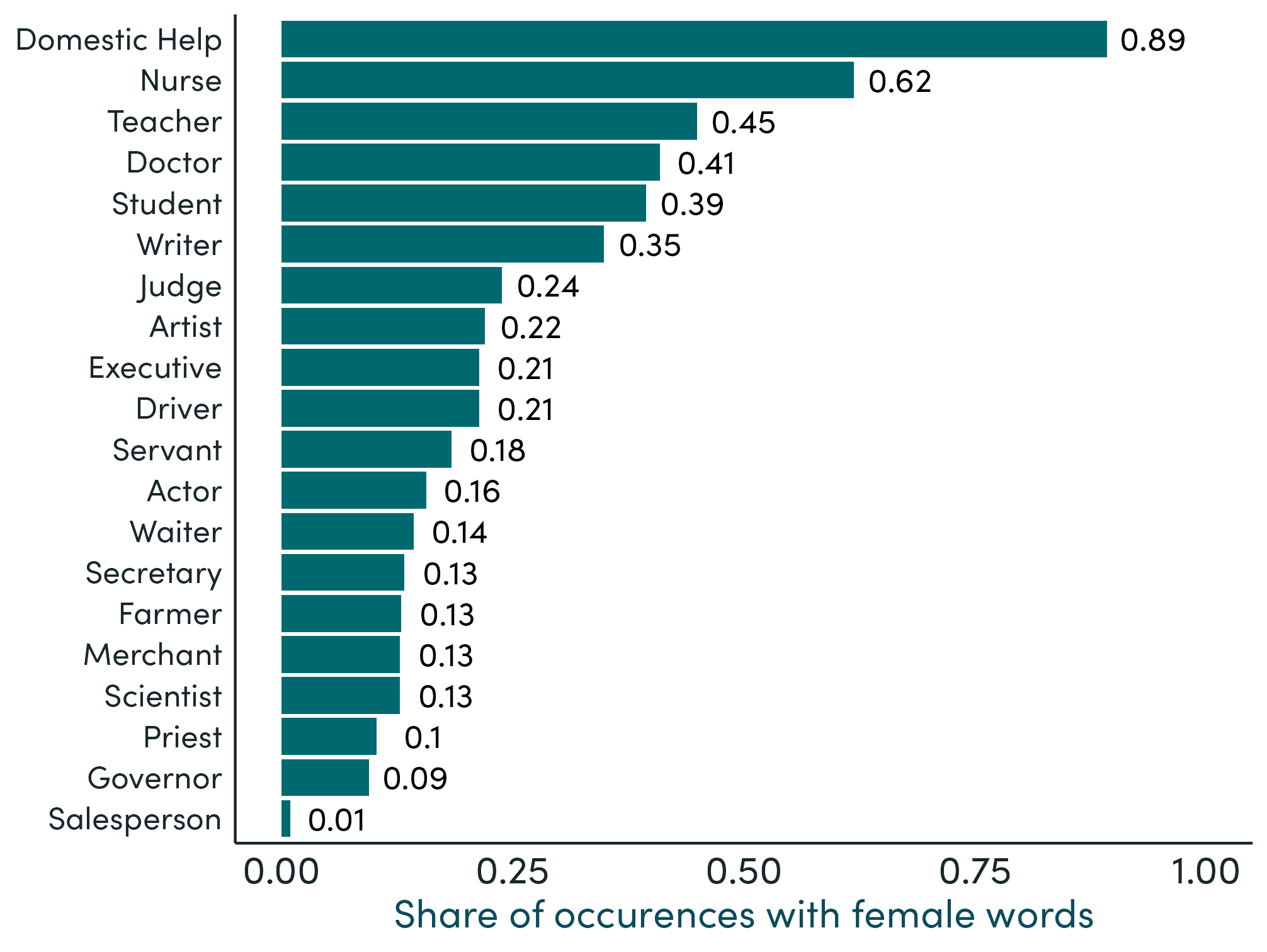

Supplement: S9 Fig — Note: This figure lists the 15 most common occupations in our full text corpus. The x-axis shows the share of all co-occurrences of the occupation and a gendered word which are female. (TIF) [file pone.0310366.s009.tif]

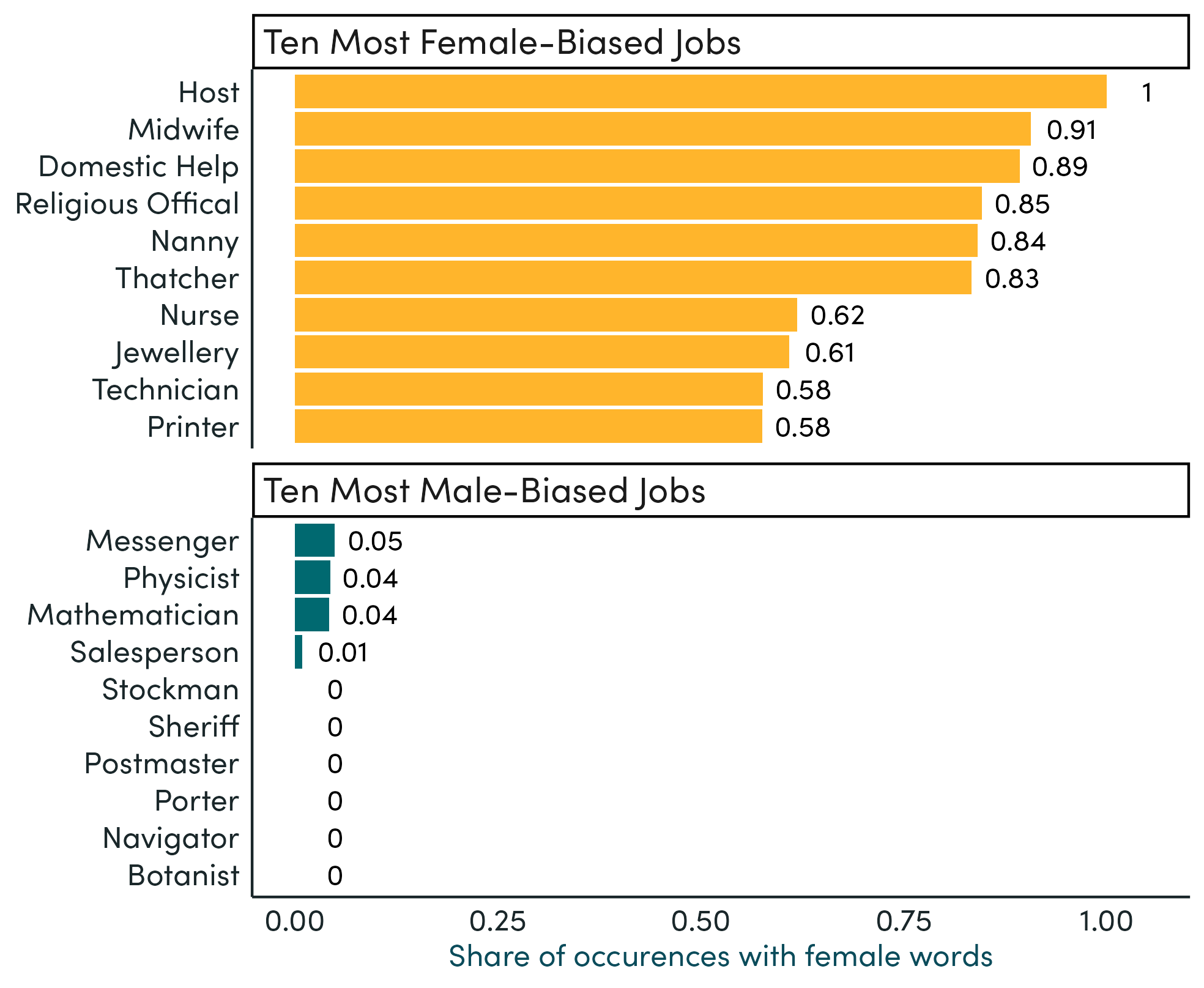

Supplement: S10 Fig — Note: This figure shows common occupation terms—classified as those with more than 10 occurrences across the corpus—which were most biased towards either gender, in the sense of co-occurring (within sentences) more frequently with terms of one than the other. (TIF) [file pone.0310366.s010.tif]

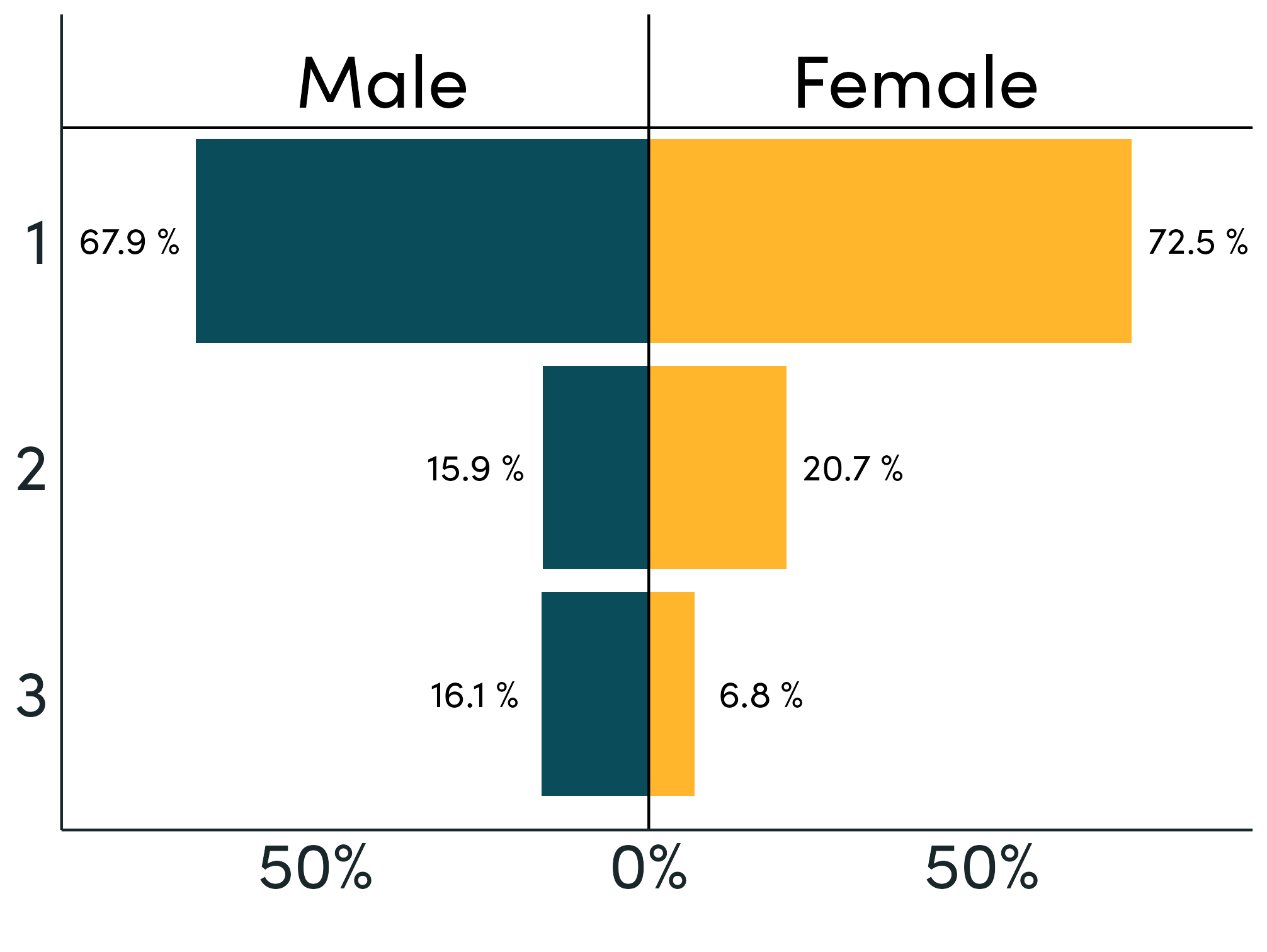

Supplement: S11 Fig — Note: This figure shows the distribution of occupation categories across co-occurrences with gendered words. That is, for all co-occurrences between a male gendered word and an occupation, 64.8 percent of those co-occurrences were with managerial or professional occupations (category 1). 13.7 percent with service (category 2) occupations, and 21.4 percent with manual (category 3) occupations. (TIF) [file pone.0310366.s011.tif]

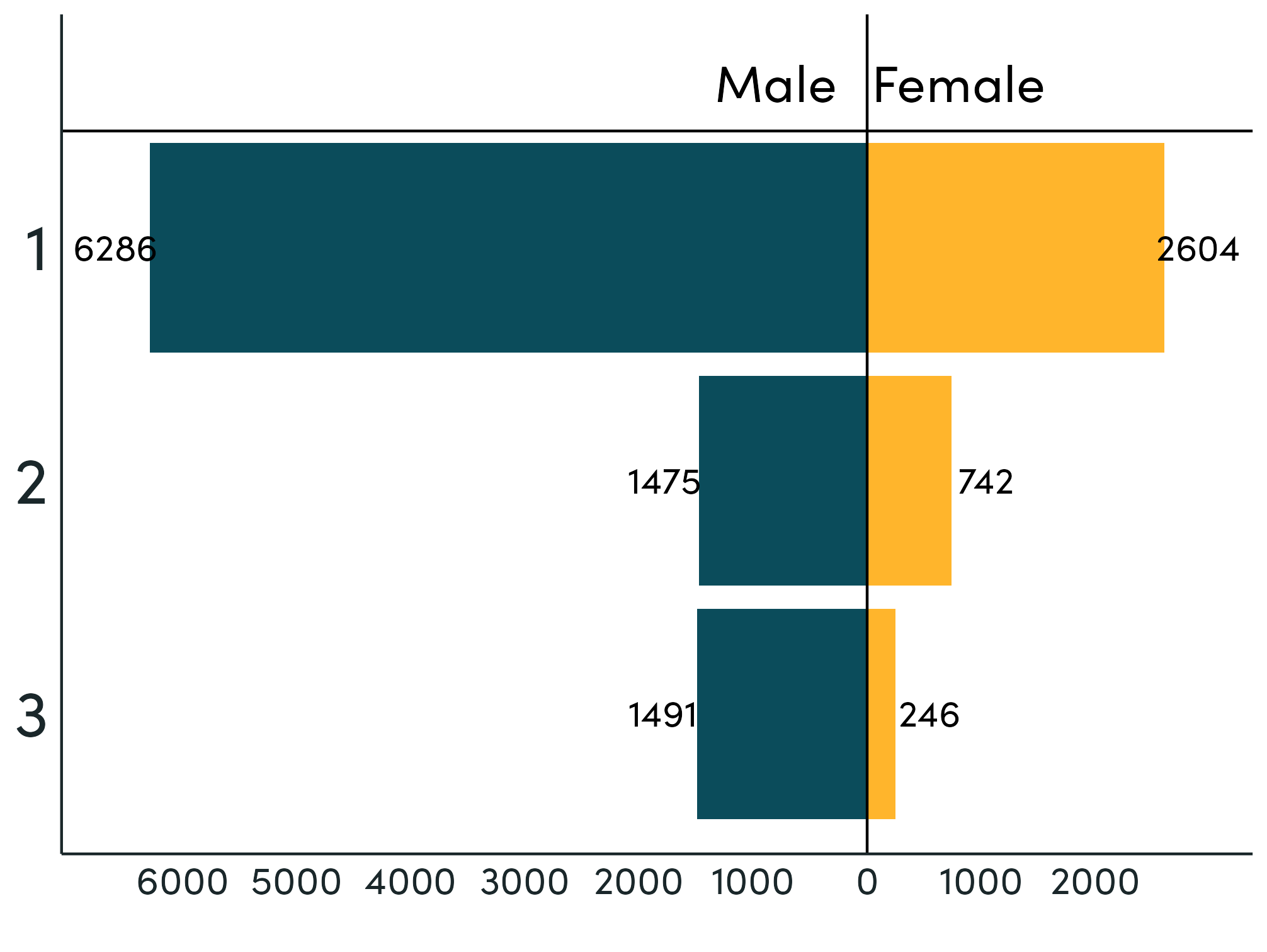

Supplement: S12 Fig — Note: This figure shows the distribution of occupation categories across co-occurrences with gendered words. In this case, absolute values are shown. For example, there were 3204 co-occurrences between male gendered words with a managerial or professional (category 1) occupation. (TIF) [file pone.0310366.s012.tif]

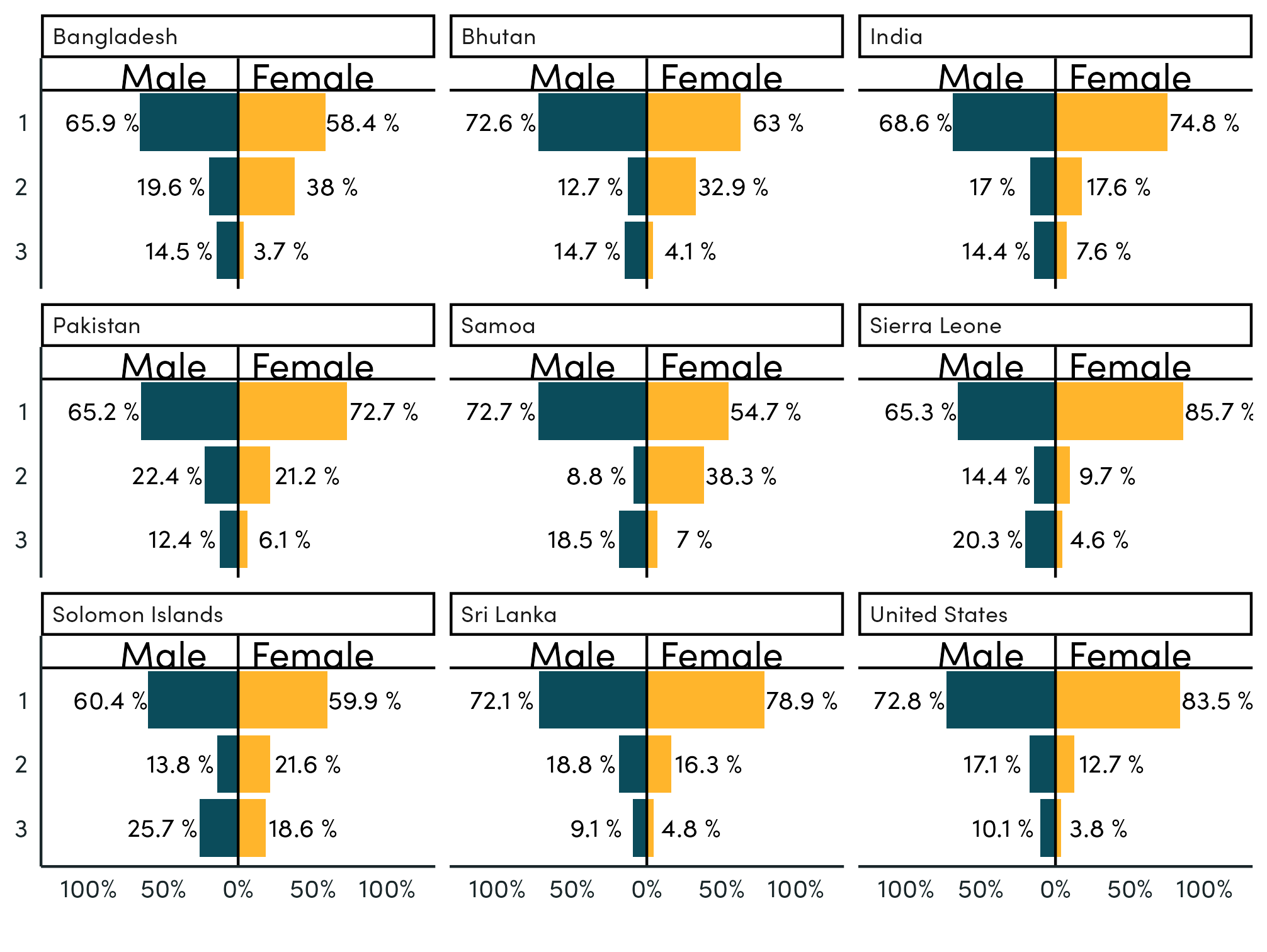

Supplement: S13 Fig — Note: This shows the results described above for the nine countries with over 250 occupation term-gender term cooccurences. (TIF) [file pone.0310366.s013.tif]

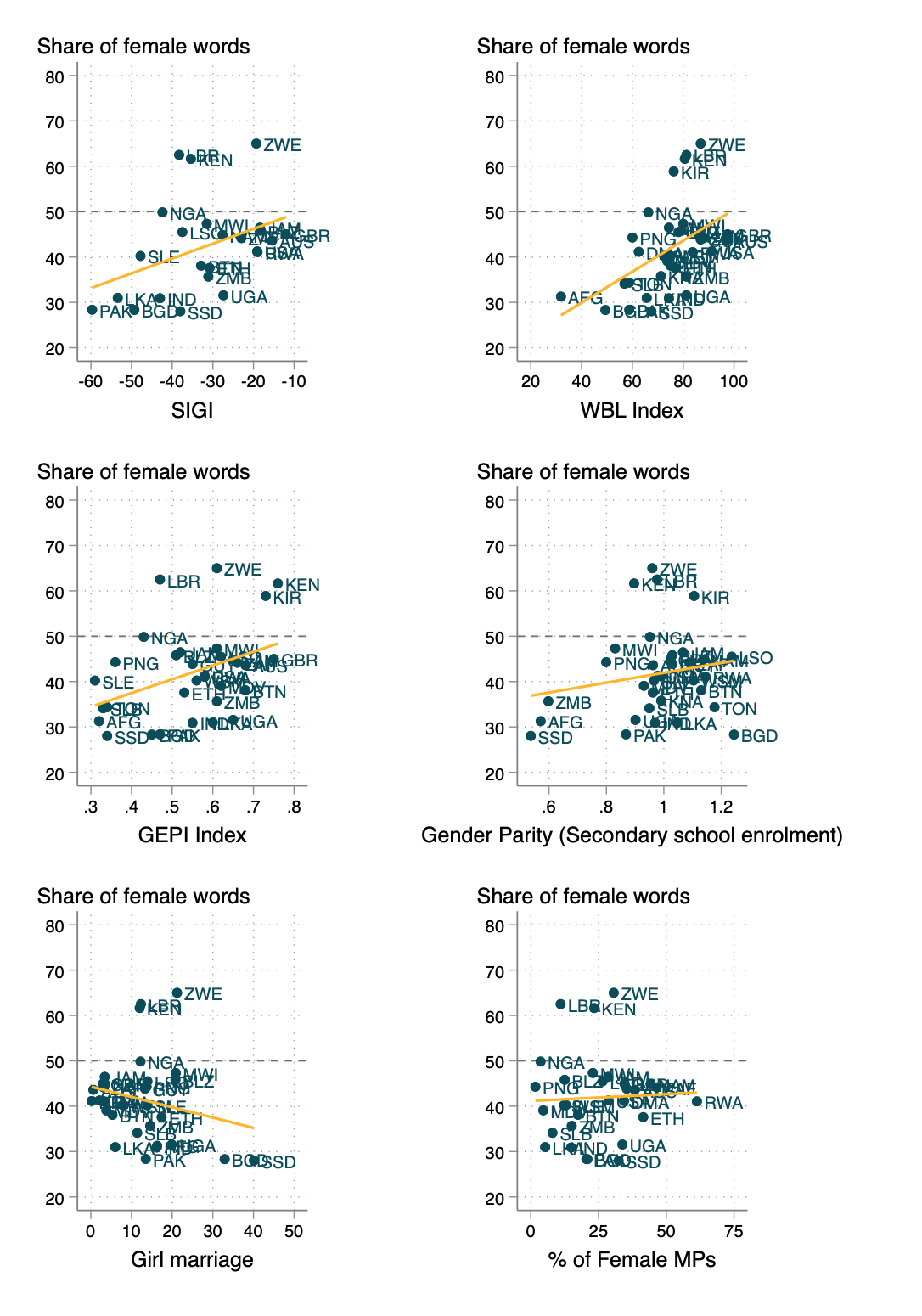

Supplement: S14 Fig — Note: This figure shows correlations between gendered word representation and country level indicators of gender equality. SIGI is the OECD Social Institutions & Gender Index. WBL is the WB Women, Business, & the Law Index. GEPI is CGD’s Girl’s Education Policy Index. Secondary Education Parity is from UNESCO. Girl marriage and female MPs are from the OECD Gender, Institutions, & Development database. (TIF) [file pone.0310366.s014.tif]

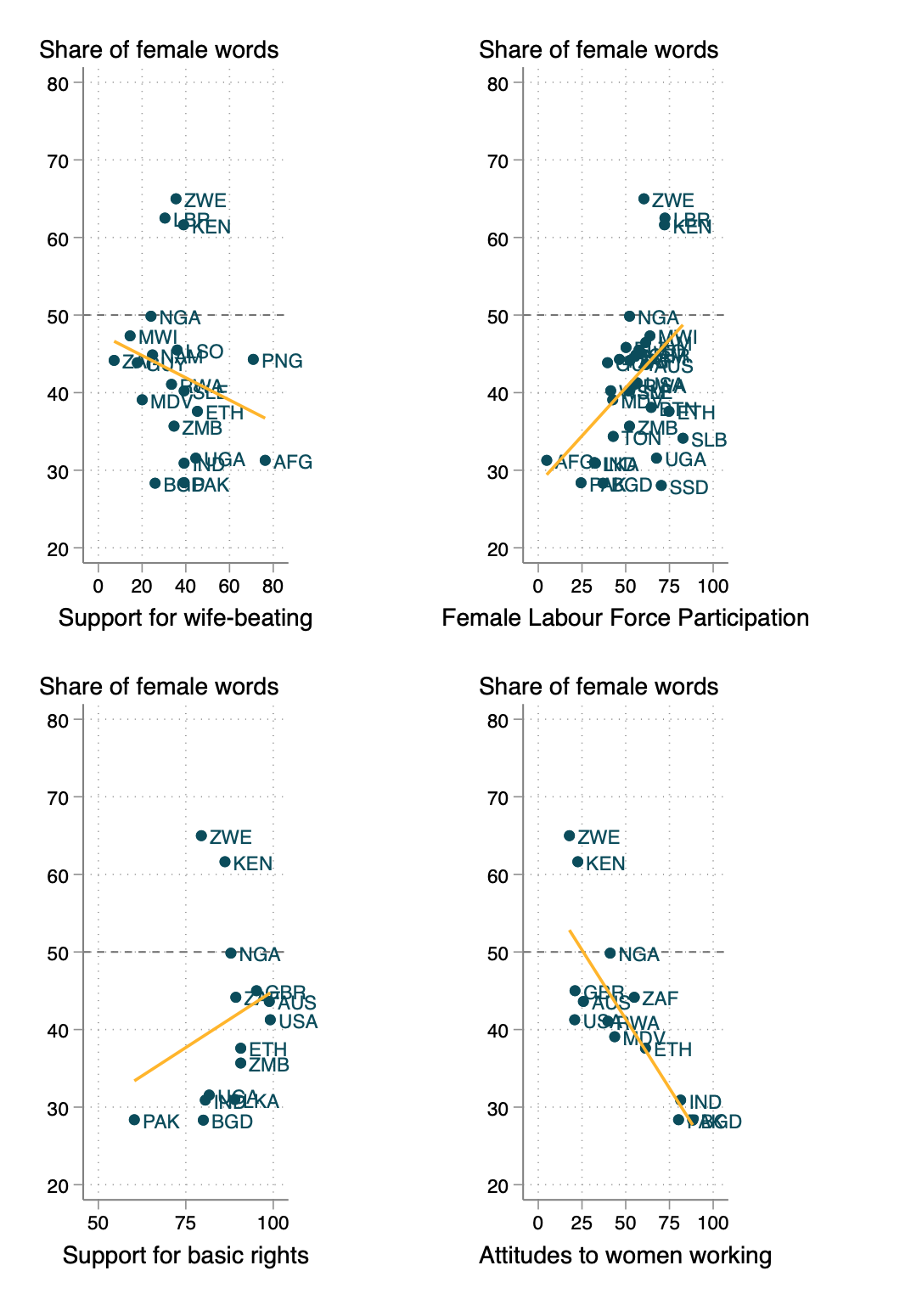

Supplement: S15 Fig — Note: This figure shows correlations between gendered word representation and country level indicators of gender equality. Support for wife-beating is from the Demographic & Health Survey. Female labour force participation is from the WB World Development Indicators. Support for basic rights and attitudes to women working are from the Gallup World Poll. (TIF) [file pone.0310366.s015.tif]

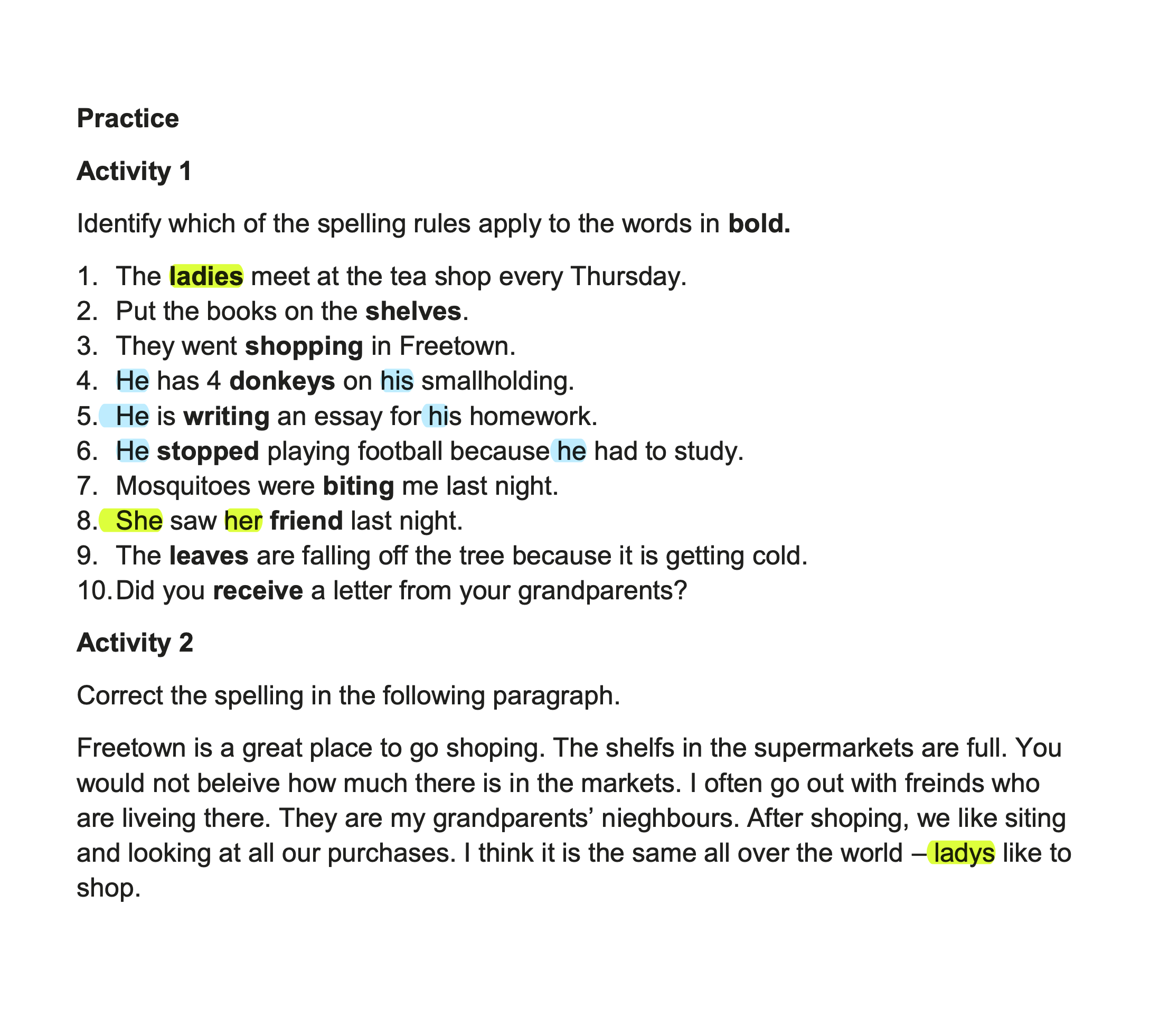

Supplement: S4 File — Note: In this sample page from a textbook we can see an uneven representation of male and female nouns and pronouns—there are 6 male themed words (he/his) and 4 female themed words (ladies/she/her/ladys). (TIF) [file pone.0310366.s019.tif]

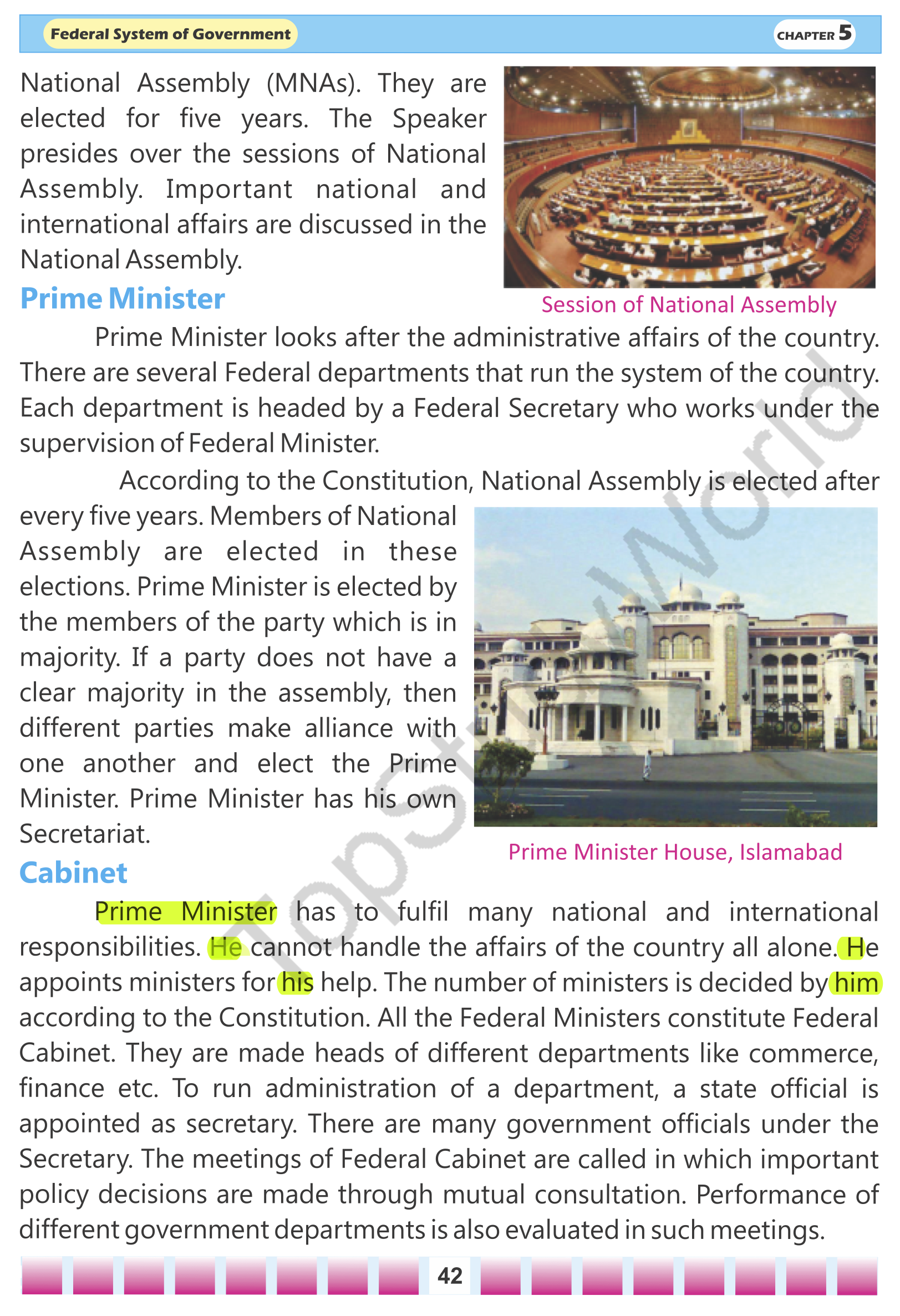

Supplement: S5 File — Note: In this sample page we see the Prime Minister being discussed as He/him/his. The current Prime Minister does happen to be a man, but he is not named in this book, and the role is clearly not inherently gendered. (TIF) [file pone.0310366.s020.tif]

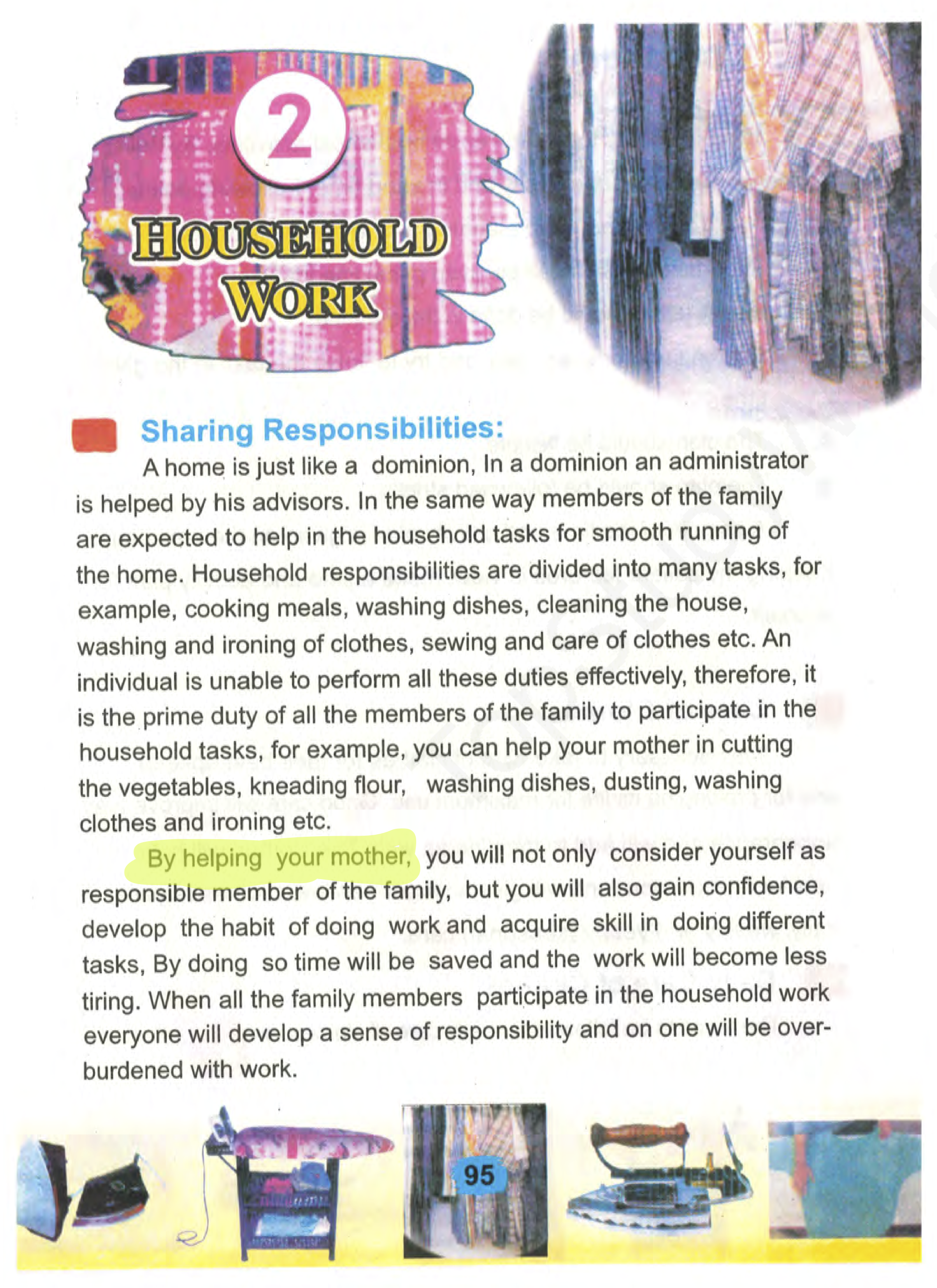

Supplement: S6 File — Note: This book discusses how “you can help your mother” with housework. (TIF) [file pone.0310366.s021.tif]
