# Supplementary material for: Sexist textbooks: Automated analysis of gender bias in 1,255 books from 34 countries
Source: PLoS One. 2024 Oct 9;19(10):e0310366. doi: 10.1371/journal.pone.0310366 (PMC11463758; doi:10.1371/journal.pone.0310366)
Supplement: S1 File — A list of all male and female gendered nouns and pronouns for which we search. (DOCX) [file pone.0310366.s016.docx]

Gendered word lists

The table below includes our list of gendered words, from Adukia et al (2023). It also codes whether words denote a young or old, or age-neutral character; and whether words were also included in gendered word lists from Lucy et al (2020) or Caliskan et al (2022). Note that this does not include the list of gendered names, which were also used to identify occurrences of male and female characters.

| **Word** | **Gender** | **Age** | **Lucy et al** | **Caliskan et al** |
| --- | --- | --- | --- | --- |
| Actress | Female |  |  |  |
| Aunt | Female | Old |  |  |
| Auntie | Female | Old |  |  |
| Aunties | Female | Old |  |  |
| Aunts | Female | Old |  |  |
| Aunty | Female | Old |  |  |
| Damsel | Female | Young |  |  |
| Damsels | Female | Young |  |  |
| Daughter | Female | Young |  | Yes |
| Daughters | Female | Young |  |  |
| Emperess | Female | Old |  |  |
| Emperesses | Female | Old |  |  |
| Empress | Female | Old |  |  |
| Empresses | Female | Old |  |  |
| Female | Female |  | Yes | Yes |
| Females | Female |  |  |  |
| Girl | Female | Young |  | Yes |
| Girls | Female | Young |  |  |
| Grandma | Female | Old |  |  |
| Grandmas | Female | Old |  |  |
| Grandmom | Female | Old |  |  |
| Grandmother | Female | Old |  |  |
| Grandmothers | Female | Old |  |  |
| Granny | Female | Old |  |  |
| Her | Female |  | Yes | Yes |
| Hers | Female |  | Yes | Yes |
| Herself | Female |  |  |  |
| Ladies | Female |  |  |  |
| Lady | Female |  |  |  |
| Madam | Female | Old |  |  |
| Madame | Female | Old |  |  |
| Maid | Female |  |  |  |
| Maiden | Female | Young |  |  |
| Maidens | Female | Young |  |  |
| Maids | Female |  |  |  |
| Mermaid | Female |  |  |  |
| Mermaids | Female |  |  |  |
| Miss | Female | Young |  |  |
| Mom | Female | Old |  |  |
| Mommies | Female | Old |  |  |
| Mommy | Female | Old |  |  |
| Moms | Female | Old |  |  |
| Mother | Female | Old |  |  |
| Mothers | Female | Old |  |  |
| Mrs | Female | Old |  |  |
| Ms | Female |  |  |  |
| Nana | Female | Old |  |  |
| Nanas | Female | Old |  |  |
| Princess | Female | Young |  |  |
| Princesses | Female | Young |  |  |
| Queen | Female | Old |  |  |
| Queens | Female | Old |  |  |
| She | Female |  | Yes | Yes |
| Sister | Female |  |  | Yes |
| Sisters | Female |  |  |  |
| Stepmother | Female | Old |  |  |
| Stepmothers | Female | Old |  |  |
| Waitress | Female |  |  |  |
| Wife | Female | Old |  |  |
| Witch | Female | Old |  |  |
| Witches | Female | Old |  |  |
| Wives | Female | Old |  |  |
| Woman | Female | Old | Yes | Yes |
| Women | Female | Old | Yes |  |
| Actor | Male |  |  |  |
| Boy | Male | Young |  | Yes |
| Boys | Male | Young |  |  |
| Bro | Male |  |  |  |
| Brother | Male |  |  | Yes |
| Brothers | Male |  |  |  |
| Chap | Male |  |  |  |
| Chaps | Male |  |  |  |
| Dad | Male | Old |  |  |
| Daddies | Male | Old |  |  |
| Daddy | Male | Old |  |  |
| Dads | Male | Old |  |  |
| Emperor | Male | Old |  |  |
| Emperors | Male | Old |  |  |
| Father | Male | Old |  |  |
| Fathers | Male | Old |  |  |
| Fellow | Male |  |  |  |
| Fellows | Male |  |  |  |
| Gentleman | Male | Old |  |  |
| Gentlemen | Male | Old |  |  |
| Granddad | Male | Old |  |  |
| Granddads | Male | Old |  |  |
| Grandfather | Male | Old |  |  |
| Grandfathers | Male | Old |  |  |
| Grandpa | Male | Old |  |  |
| Grandpas | Male | Old |  |  |
| He | Male |  | Yes | Yes |
| Him | Male |  | Yes | Yes |
| Himself | Male |  |  |  |
| His | Male |  | Yes | Yes |
| Hisself | Male |  |  |  |
| Husband | Male | Old |  |  |
| Husbands | Male | Old |  |  |
| King | Male | Old |  |  |
| Kings | Male | Old |  |  |
| Knight | Male | Old |  |  |
| Lad | Male | Young |  |  |
| Lads | Male | Young |  |  |
| Lord | Male | Old |  |  |
| Lords | Male | Old |  |  |
| Male | Male |  | Yes | Yes |
| Males | Male |  |  |  |
| Man | Male | Old | Yes | Yes |
| Master | Male | Young |  |  |
| Masters | Male | Young |  |  |
| Men | Male | Old | Yes |  |
| Merman | Male |  |  |  |
| Mermen | Male |  |  |  |
| Mr | Male | Old |  |  |
| Paige | Male | Young |  |  |
| Paiges | Male | Young |  |  |
| Papa | Male | Old |  |  |
| Papas | Male | Old |  |  |
| Prince | Male | Young |  |  |
| Princes | Male | Young |  |  |
| Sir | Male | Old |  |  |
| Sirs | Male | Old |  |  |
| Son | Male | Young |  | Yes |
| Sons | Male | Young |  |  |
| Squire | Male | Young |  |  |
| Squires | Male | Young |  |  |
| Stepfather | Male | Old |  |  |
| Stepfathers | Male | Old |  |  |
| Uncle | Male | Old |  |  |
| Uncles | Male | Old |  |  |
| Waiter | Male |  |  |  |
| Wizard | Male |  |  |  |
| Wizards | Male |  |  |  |
| Mum | Female | Old |  |  |
| Mummies | Female | Old |  |  |
| Mummy | Female | Old |  |  |
| Mums | Female | Old |  |  |
|  |  |  |  |  |
|  |  |  |  |  |
|  |  |  |  |  |
|  |  |  |  |  |
