# Supplementary material for: Sexist textbooks: Automated analysis of gender bias in 1,255 books from 34 countries
Source: PLoS One. 2024 Oct 9;19(10):e0310366. doi: 10.1371/journal.pone.0310366 (PMC11463758; doi:10.1371/journal.pone.0310366)
Supplement: S2 File — A list of all occupations for which we search. (DOCX) [file pone.0310366.s017.docx]

Occupation job type word list

The table below includes our list of occupation words with coded job type.

| **Occupation** | **ISCO-08** |
| --- | --- |
| actress | 1 |
| translator | 1 |
| astronomer | 1 |
| butler | 2 |
| counsellor | 1 |
| economist | 1 |
| illustrator | 1 |
| interpreter | 1 |
| landlord | 1 |
| envoy | 1 |
| potter | 3 |
| receptionist | 2 |
| researcher | 1 |
| steward | 2 |
| builder | 3 |
| buyer | 1 |
| cleaner | 2 |
| preacher | 1 |
| proprietor | 1 |
| sculptor | 1 |
| sportsman | 1 |
| housewife | 2 |
| supervisor | 1 |
| tutor | 1 |
| blacksmith | 3 |
| consultant | 1 |
| housekeeper | 2 |
| treasurer | 1 |
| weaver | 2 |
| carpenter | 2 |
| hawker | 3 |
| surveyor | 1 |
| attendant | 2 |
| biologist | 1 |
| cashier | 2 |
| foreman | 2 |
| librarian | 1 |
| instructor | 1 |
| postman | 2 |
| historian | 1 |
| miller | 3 |
| nanny | 2 |
| baker | 3 |
| dealer | 2 |
| goldsmith | 3 |
| interviewer | 1 |
| inventor | 1 |
| musician | 1 |
| operator | 2 |
| surgeon | 1 |
| nun | 1 |
| operative | 2 |
| gardener | 3 |
| mechanic | 3 |
| magistrate | 1 |
| architect | 1 |
| photographer | 1 |
| salesman | 2 |
| chemist | 1 |
| journalist | 1 |
| reporter | 1 |
| lecturer | 1 |
| mathematician | 1 |
| inspector | 1 |
| labourer | 3 |
| physicist | 1 |
| shepherd | 3 |
| accountant | 1 |
| administrator | 1 |
| porter | 3 |
| producer | 1 |
| barber | 2 |
| clerk | 2 |
| editor | 1 |
| physician | 1 |
| publisher | 1 |
| tailor | 2 |
| engineer | 1 |
| lawyer | 1 |
| magician | 1 |
| executive | 1 |
| messenger | 3 |
| pilot | 1 |
| soldier | 3 |
| waiter | 2 |
| scientist | 1 |
| nurse | 1 |
| secretary | 2 |
| maid | 2 |
| judge | 1 |
| merchant | 1 |
| actor | 1 |
| governor | 1 |
| artist | 1 |
| driver | 3 |
| writer | 1 |
| priest | 1 |
| servant | 2 |
| farmer | 3 |
| doctor | 1 |
| teacher | 1 |
