# Supplementary material for: Sexist textbooks: Automated analysis of gender bias in 1,255 books from 34 countries
Source: PLoS One. 2024 Oct 9;19(10):e0310366. doi: 10.1371/journal.pone.0310366 (PMC11463758; doi:10.1371/journal.pone.0310366)
Supplement: S3 File — A list of thematic words related to ‘home’, ‘achievement’, ‘appearance’, and ‘work’. (DOCX) [file pone.0310366.s018.docx]

Theme word list

The table below includes our list of theme words, gathered from Lucy et al. (2020), Adukia et al. (2022), and Kumar et al. (2021).

| **Word** | **Theme** | **Source** |
| --- | --- | --- |
| achievement | Achievement | Lucy et al. (2020) |
| authority | Achievement | Lucy et al. (2020) |
| better | Achievement | Lucy et al. (2020) |
| control | Achievement | Lucy et al. (2020) |
| effort | Achievement | Lucy et al. (2020) |
| leader | Achievement | Lucy et al. (2020) |
| plan | Achievement | Lucy et al. (2020) |
| persuasive | Achievement | Adukia et al |
| power | Achievement | Lucy et al. (2020) |
| powerful | Achievement | Lucy et al. (2020) |
| success | Achievement | Lucy et al. (2020) |
| tried | Achievement | Lucy et al. (2020) |
| won | Achievement | Lucy et al. (2020) |
| alluring | Appearance | Adukia et al |
| elegant | Appearance | Adukia et al |
| beautiful | Appearance | Adukia et al |
| slim | Appearance | Adukia et al |
| ugly | Appearance | Adukia et al |
| gorgeous | Appearance | Adukia et al |
| children | Family | Kumar et al |
| chore | Family | Lucy et al. (2020) |
| cousins | Family | Kumar et al |
| domestic | Family | Lucy et al. (2020) |
| family | Family | Kumar et al / Lucy et al. (2020) |
| home | Family | Kumar et al / Lucy et al. (2020) |
| household | Family | Lucy et al. (2020) |
| marriage | Family | Kumar et al |
| parents | Family | Kumar et al |
| relatives | Family | Kumar et al |
| wedding | Family | Kumar et al |
| business | Work | Lucy et al. (2020) |
| career | Work | Kumar et al |
| company | Work | Lucy et al. (2020) |
| corporation | Work | Kumar et al |
| economy | Work | Lucy et al. (2020) |
| executive | Work | Kumar et al |
| industry | Work | Lucy et al. (2020) |
| job | Work | Lucy et al. (2020) |
| labor | Work | Lucy et al. (2020) |
| labour | Work | Lucy et al. (2020) |
| management | Work | Kumar et al |
| office | Work | Kumar et al |
| pay | Work | Lucy et al. (2020) |
| professional | Work | Kumar et al |
| salary | Work | Lucy et al. (2020) |
| trade | Work | Lucy et al. (2020) |
| wage | Work | Lucy et al. (2020) |
| work | Work | Lucy et al. (2020) |
| workers | Work | Lucy et al. (2020) |
| working | Work | Lucy et al. (2020) |
