# Supplementary material for: Sexist textbooks: Automated analysis of gender bias in 1,255 books from 34 countries
Source: PLoS One. 2024 Oct 9;19(10):e0310366. doi: 10.1371/journal.pone.0310366 (PMC11463758; doi:10.1371/journal.pone.0310366)
Supplement: S1 Table — (DOCX) [file pone.0310366.s023.docx]

Gender equality in countries in our sample vs the rest of the world

|  | Our sample | Other Countries |
| --- | --- | --- |
| GDP per capita ($) | 9247 | 22982 |
| SIGI Index | -32 | -28 |
| WBL Index | 74 | 78 |
| GEPI Index | 54 | 62 |
| Sec Ed Parity Index | 98 | 100 |
| Girl Marriage (%) | 12 | 10 |
| Female MPs (%) | 24 | 27 |
