# Supplementary material for: Sexist textbooks: Automated analysis of gender bias in 1,255 books from 34 countries
Source: PLoS One. 2024 Oct 9;19(10):e0310366. doi: 10.1371/journal.pone.0310366 (PMC11463758; doi:10.1371/journal.pone.0310366)
Supplement: S2 Table — Note: This table shows the sources and publishing information for the textbooks included in our corpus. (DOCX) [file pone.0310366.s024.docx]

Book sources

| **Country** | **Policy on Textbook Selection** | **Source Type** | **Link 1** | **Publisher Type** | **Publisher** | **External agency funding** | **Resource Type** |
| --- | --- | --- | --- | --- | --- | --- | --- |
| Afghanistan | Government-approved | Government website | https://moe.gov.af/ | Government | Ministry of Education | | Student's book |
| Australia | School Autonomy | Other | https://pascalpress.com.au/free-to-download-resources/ | Private | Pascal Press | | Workbooks |
| Bangladesh | Government-approved | Government website | http://www.nctb.gov.bd/site/page/85da3a9e-fd7f-4b2f-be74-f8e6637ae785/- | Government | National Curriculum and Textbook Board | | Textbook |
| Belize | School Autonomy | Government website | https://www.moecst.gov.bz/ | Government | Ministry of Education | | Workbooks |
| Bhutan | Government-approved | Government website | http://www.education.gov.bt/?p=4163 | Government | Ministry of Education | Canadian International Development Agency | Self-Instructional Materials |
| Canada |  |  |  |  |  |  |  |
| Dominica | School Autonomy | Government website | http://education.gov.dm/support-material | Government | Ministry of Education | | Workbooks |
| Ethiopia | Government-approved | Government website | https://www.neaea.com/ethiopian-grade-7-textbook/ | Government | Ministry of Education | Governments of Finland, Italy, Netherlands and the United Kingdom. | Textbook |
| Guyana | School Autonomy | Government website | www.education.gov.gy | Government | Ministry of Education | Inter-American Development Bank | Textbook |
| India | Mixed | Other | https://byjus.com/ncert-books/ | Government | National Council of Educational Research and Training | | Textbook |
| Jamaica | School Autonomy | Government website | https://www.google.com/url?q=https://moey.gov.jm/mathematics&sa=D&source=editors&ust=1697537562276134&usg=AOvVaw1EFIRurHZOMdWA-TYnKnqV | Government | Ministry of Education | | Workbooks |
| Kenya | Government-approved | Other | pdf.usaid.gov | Government | Ministry of Education | USAID, RTI | |
| Kiribati | Government-approved | Government website | https://drive.google.com/drive/u/0/folders/1v5nF_jfqXdcG4Xlez2H1tFvCnWmbmOf_?fbclid=IwAR3Qwmj2Eej-xhhymSM-Ufabl354SjJWt3nmSPuzKiFp0zMKX2obfjYixh4 | Government | Ministry of Education | |  |
| Lesotho | Government-approved | Other | https://oasis.col.org/communities/208bd608-9549-44ec-9546-92ce3c2247cb | Government | Ministry of Education | Commonwealth of Learning | |
| Liberia | Government-approved | Other | http://docplayer.net/145378611-Ministry-of-education.html | Government | Ministry of Education | USAID |  |
| Maldives | Government-approved | Government website | [https://www.nie.edu.mv/index.php/en/national-curriculum/student-books; https://www.nie.edu.mv/index.php/en/](https://www.nie.edu.mv/index.php/en/national-curriculum/student-books;) | Government | National Institute of Education, Cambridge University Press | | |
| Namibia | School Autonomy | Other | https://freekidsbooks.org/open-educational-resources/ | Government | Ministry of Education | Commonwealth of Learning | Textbook and Self study learning |
| Nigeria | Government-approved | Other | pdf.usaid.gov | Government | Ministry of Education | USAID |  |
| Pakistan | Mixed | Other | [www.topstudyworld.com/books; https://ebooks.stbb.edu.pk/](http://www.topstudyworld.com/books;) | Government, Private | Ch. Ghulam Rasul and Sons, Punjab Curriculum Board, Sindh Curriculum Board | | |
| Papua New Guinea | Government-approved | Government website | https://www.education.gov.pg/quicklinks/gr1-6-maths-science-textbooks.html | Government | Department of Education | Japan | Textbook |
| Rwanda | Government-approved | Government website | https://elearning.reb.rw/course | Government, Private | Rwanda Education Board / Longhorn | | |
| Samoa | Government-approved | Government website | https://www.mesc.gov.ws/education/textbooks/ | Government | Ministry of Education, Sports and Culture | NZAID | Textbook |
| Seychelles | Government-approved | Other | https://oasis.col.org/communities/208bd608-9549-44ec-9546-92ce3c2247cb | Government | Ministry of Education | Commonwealth of Learning | |
| Sierra Leone | Government-approved | Government website | https://mbsseknowledgeplatform.gov.sl/materials/ | Government | Ministry of Basic and Senior Secondary Education | UKAID; Cambridge Education; International Rescue Committee | Pupil Handbooks |
| Solomon Islands | Government-approved | Government website | http://www.iresource.gov.sb/ | Government | Ministry of Education and Human Resource Development | | |
| South Africa | School Autonomy | Both | https://www.education.gov.za/Curriculum/LearningandTeachingSupportMaterials(LTSM)/Workbooks.aspx; https://www.siyavula.com/read | Government | Department of Basic Education / Siyavula | USAID | Workbooks |
| South Sudan | Government-approved | Other | https://cgatechnologies.org.uk/teaching-and-learning-materials | Government | Ministry of General Education and Instruction | GPE | Pupil's Book |
| Sri Lanka | Government-approved | Government website | http://www.edupub.gov.lk/BooksDownload.php | Government | Ministry of Education | | Pupil's Book |
| St Kitts and Nevis | School Autonomy | Government website | https://www.education.gov.kn/ | Government | Ministry of Education | | Workbooks |
| Tonga | Government-approved | Government website | https://pacificschoolserver.org/content/481 | Government, Foreign agency | Secretariat of the Pacific Community/United States Department of Agriculture | United States Department of Agriculture | |
| Trinidad and Tobago | Government-approved | Other | https://oasis.col.org/communities/208bd608-9549-44ec-9546-92ce3c2247cb | Government | Ministry of Education | Commonwealth of Learning | |
| Uganda | Government-approved | Government website | https://www.ncdc.go.ug/resources#sppb-tab-1688120906302 | Government | National Curriculum Development Center | | Self-study learning |
| United Kingdom | School Autonomy | Other |  | Private | Collins |  | Pupil Books |
| United States | School Autonomy | Other | https://www.loadsoflearning.com/free-complete-textbook-series-pdfs | Private | Macmillan McGraw | |  |
| Zambia | Government-approved | Government website | https://www.moge.gov.zm/ | Government | Commonwealth of Learning/Curriculum Development Center | Commonwealth of Learning | |
| Zimbabwe | Government-approved | Government website | mopse.co.zw | Government | Ministry of Primary and Secondary Education | Girl's Education Challenge - UKAID | |
