# Supplementary material for: Sexist textbooks: Automated analysis of gender bias in 1,255 books from 34 countries
Source: PLoS One. 2024 Oct 9;19(10):e0310366. doi: 10.1371/journal.pone.0310366 (PMC11463758; doi:10.1371/journal.pone.0310366)
Supplement: Attachment — Submitted filename: Response to Reviewers (Sexist Textbooks).docx [file pone.0310366.s025.docx]

Dear Johannes,

Thank you for the opportunity to revise and resubmit our manuscript “Sexist Textbooks.” We have engaged every comment and incorporated most of them. We believe that the paper is much stronger as a result.

Below we reproduce the reviewer comments *in italics* and our responses—together with the revisions that we have made—in plain text.

Warm regards,

Lee Crawfurd, Christelle Saintis-Miller, Rory Todd

**Journal Requirements:**

*1. Please ensure that your manuscript meets PLOS ONE's style requirements, including those for file naming. The PLOS ONE style templates can be found at* [*https://journals.plos.org/plosone/s/file?id=wjVg/PLOSOne_formatting_sample_main_body.pdf*](https://journals.plos.org/plosone/s/file?id=wjVg/PLOSOne_formatting_sample_main_body.pdf) *and*

[*https://journals.plos.org/plosone/s/file?id=ba62/PLOSOne_formatting_sample_title_authors_affiliations.pdf*](https://journals.plos.org/plosone/s/file?id=ba62/PLOSOne_formatting_sample_title_authors_affiliations.pdf)

We have ensured we follow these style guides.

*2. Thank you for stating the following financial disclosure: "This research was supported by funding from the Bill and Melinda Gates Foundation and Echidna Giving."*

*Please state what role the funders took in the study. If the funders had no role, please state: "The funders had no role in study design, data collection and analysis, decision to publish, or preparation of the manuscript."*

*If this statement is not correct you must amend it as needed.*

*Please include this amended Role of Funder statement in your cover letter; we will change the online submission form on your behalf.*

The funders had no role in this study. We would be grateful if you could update the submission form on our behalf to read: “The funders had no role in study design, data collection and analysis, decision to publish, or preparation of the manuscript”.

*3. Please update your submission to use the PLOS LaTeX template. The template and more information on our requirements for LaTeX submissions can be found at http://journals.plos.org/plosone/s/latex.*

We have updated our submission in the PLOS LaTeX template.

*4. Please note that in order to use the direct billing option the corresponding author must be affiliated with the chosen institute. Please either amend your manuscript to change the affiliation or corresponding author, or email us at plosone@plos.org with a request to remove this option.*

We have emailed [plosone@plos.org](mailto:plosone@plos.org) to request the removal of the direct billing option.

*5. Thank you for uploading your study's underlying data set. Unfortunately, the repository you have noted in your Data Availability statement does not qualify as an acceptable data repository according to PLOS's standards. At this time, please upload the minimal data set necessary to replicate your study's findings to a stable, public repository (such as figshare or Dryad) and provide us with the relevant URLs, DOIs, or accession numbers that may be used to access these data. For a list of recommended repositories and additional information on PLOS standards for data deposition, please see https://journals.plos.org/plosone/s/recommended-repositories.*

We have added the code and data necessary to replicate all analysis to the Harvard dataverse, a recommended repository, [here](https://dataverse.harvard.edu/dataset.xhtml?persistentId=doi:10.7910/DVN/AJD0T4&faces-redirect=true).

*6. Please amend your list of authors on the manuscript to ensure that each author is linked to an affiliation. Authors’ affiliations should reflect the institution where the work was done (if authors moved subsequently, you can also list the new affiliation stating “current affiliation:….” as necessary).*

We have amended the author details in the manuscript to reflect more clearly that all authors are affiliated with the Center for Global Development.

*7. We note that [Figure 1] in your submission contain [map/satellite] images which may be copyrighted. All PLOS content is published under the Creative Commons Attribution License (CC BY 4.0), which means that the manuscript, images, and Supporting Information files will be freely available online, and any third party is permitted to access, download, copy, distribute, and use these materials in any way, even commercially, with proper attribution. For these reasons, we cannot publish previously copyrighted maps or satellite images created using proprietary data, such as Google software (Google Maps, Street View, and Earth). For more information, see our copyright guidelines: http://journals.plos.org/plosone/s/licenses-and-copyright. We require you to either (1) present written permission from the copyright holder to publish these figures specifically under the CC BY 4.0 license, or (2) remove the figures from your submission:*

*a. You may seek permission from the original copyright holder of Figure 1 to publish the content specifically under the CC BY 4.0 license.*

*We recommend that you contact the original copyright holder with the Content Permission Form (http://journals.plos.org/plosone/s/file?id=7c09/content-permission-form.pdf) and the following text:*

*“I request permission for the open-access journal PLOS ONE to publish XXX under the Creative Commons Attribution License (CCAL) CC BY 4.0 (http://creativecommons.org/licenses/by/4.0/). Please be aware that this license allows unrestricted use and distribution, even commercially, by third parties. Please reply and provide explicit written permission to publish XXX under a CC BY license and complete the attached form.”*

*Please upload the completed Content Permission Form or other proof of granted permissions as an ""Other"" file with your submission.*

*In the figure caption of the copyrighted figure, please include the following text: “Reprinted from [ref] under a CC BY license, with permission from [name of publisher], original copyright [original copyright year].”*

*b. If you are unable to obtain permission from the original copyright holder to publish these figures under the CC BY 4.0 license or if the copyright holder’s requirements are incompatible with the CC BY 4.0 license, please either i) remove the figure or ii) supply a replacement figure that complies with the CC BY 4.0 license. Please check copyright information on all replacement figures and update the figure caption with source information. If applicable, please specify in the figure caption text when a figure is similar but not identical to the original image and is therefore for illustrative purposes only.*

*The following resources for replacing copyrighted map figures may be helpful:*

*USGS National Map Viewer (public domain): http://viewer.nationalmap.gov/viewer/*

*The Gateway to Astronaut Photography of Earth (public domain): http://eol.jsc.nasa.gov/sseop/clickmap/*

*Maps at the CIA (public domain): https://www.cia.gov/library/publications/the-world-factbook/index.html and https://www.cia.gov/library/publications/cia-maps-publications/index.html*

*NASA Earth Observatory (public domain): http://earthobservatory.nasa.gov/*

*Landsat: http://landsat.visibleearth.nasa.gov/*

*USGS EROS (Earth Resources Observatory and Science (EROS) Center) (public domain): http://eros.usgs.gov/#*

*Natural Earth (public domain): http://www.naturalearthdata.com/"*

Figure 1 was generated by the authors using the online visualization tool Datawrapper. According to Datawrapper's guidelines, we have full copyright to any charts/maps created. We can therefore affirm that we have the right to use the figure and are happy for it to be published under CC BY 4.0 along with the rest of the paper.

**Reviewer #1:**

*"This is an incredibly interesting paper and I wholeheartedly support this type of work. My main criticisms of the work pertain to the authors’ use of text processing methods. I’ve roughly organized my comments in the order of which the paper is also organized.*

*The authors should re-examine possible mismatches between their methodological approaches and their end goal of measuring “stereotypes” in text. For example, one of the weaknesses of using older, widely-used lexicons such as the Valence, Arousal, and Dominance (VAD) lexicon to measure stereotypes is that they were not originally intended for stereotype measurement. That is, how does valence or arousal actually relate to gender stereotypes? There is a large amount of psychological theory around what a stereotype is and how they may be operationalized, and also growing work in text processing that centers theory-driven frameworks. I suggest the authors look towards approaches outlined in “Theory-Grounded Measurement of U.S. Social Stereotypes in English Language Models” by Cao et al. 2022 in NAACL and “Understanding and Countering Stereotypes: A Computational Approach to the Stereotype Content Model” by Fraser et al. 2021 in ACL. I understand that many of the approaches in this paper borrow methods from work such as Adukia et al. 2023 and Lucy et al. 2020, which tend to have a “everything and the kitchen sink” methodological approach to analyzing school curriculum. For Lucy et al. 2020, this may have been fine because their main aim was to demonstrate the use of NLP (natural language processing) methods on textbook data, rather than the findings themselves. This current paper, as it recycles many tried-and-true text analysis approaches, may find that its more valuable contribution is around its findings, and so it should select its methods more carefully."*

Thank you for this excellent feedback. We have explored the approaches outlined in Cao et al and Fraser et al. One of the strengths of the VAD lexicon is that Mohammad (2018) uses human raters to provide quantitative scores for a large set of 20,000 words, ensuring a high degree of overlap with our corpus. The framework in Cao et al has only 72 words, and in Fraser et al only 235 words. Only 47 of the 72 words from Cao et al occur at least once in our corpus of books, and only 148 of the 235 words from Fraser et al. The majority of books in our corpus have no mention of any of the words in the Cao et al and Fraser et al frameworks. Though the VAD lexicon was not designed explicitly for measuring stereotypes, it has been used for measuring gender bias in several contexts - for example [Martinkova et al 2023 in ACL](https://aclanthology.org/2023.bsnlp-1.17/) and [Caliskan et al 2022 in AIES](https://arxiv.org/pdf/2206.03390.pdf). There also seems to be some degree of conceptual overlap between the ‘valence’ from the VAD framework, ‘warmth’ in Fraser et al, and ‘communion’ in Cao et al. Similarly between ‘arousal’ and ‘dominance’ in the VAD framework, ‘competence’ in Fraser et al, and ‘agency’ in Cao et al. We have therefore kept the reporting of the aggregate gender difference in valence, arousal, and dominance, but removed Table 3, and included further disaggregated analysis as suggested by reviewer 2 in the supplementary material.

*I am not sure why the authors employ both word co-occurrence and cosine similarity of word embeddings in this paper, as word embeddings are learned from word co-occurrences. What does one method offer that the other does not?*

We have added the following paragraph on page 8 giving an overview of the methods we use to look at stereotypes and why: "We use three different methods to assess gender stereotypes. The simplest of these is simply counting word co-occurrences within sentences. Second, we use word embeddings which are numerical representations of words to quantitatively measure the similarity between gendered words and other thematic words. Third is a method known as ‘dependency parsing', in which we infer the relationships between words and identify the specific verbs and adjectives used alongside gendered nouns. Using a variety of methods increases our confidence in the results and aids understanding."

*In addition, at the end of section 3.3.2., the authors write “We calculate the similarity of embeddings as their cosine product.” What is a “cosine product”? Similarity of embeddings is usually calculated as cosine similarity, which is the dot product of two vectors divided by the product of their magnitudes.*

This was indeed an error which we have corrected - it should have read 'cosine similarity' and not cosine product.

*Section 3.3.3: the section is titled as “part of speech tagging” but the description seems more like dependency parsing: “see how male and female characters are depicted, what actions they are depicted doing, and how active or passive they are (for instance depending on whether they are the subject or object of sentences)”. Please clarify whether this approach is actually part of speech tagging, or if it’s actually dependency parsing.*

In the paper we use both part-of-speech tagging and dependency parsing. We've corrected the terminology to make sure these are distinguished.

*Section 3.2: “whether gendered words are capitalised or not, as an indication of whether characters are the active subject or passive object of a sentence”. This is a poor and noisy indication of whether characters are the active subject or passive object of a sentence. In many cases, the subject of a sentence might appear not as the first word in a sentence. Since the authors seem to be running dependency parsing already, why not use dependency parsed relations to determine whether someone is the subject or object? For example, the “nsubj” relation in UDPipe indicates that one word or token is the nominal subject of another.*

Thank you for this helpful suggestion. We have removed the analysis based on capitalisation, and instead report analysis based on the subject/object classification through dependency parsing. On page 11 we write: “Part-of-speech tagging shows no meaningful difference in the proportion of male and female words that appear as the subject rather than object of the sentence: 70.9% of female words and 70.3% of male words appear as the subject.”

*Many of the results the authors show are over the entire corpus, but findings around how the provenance and subject area of data affect bias measurements are far more interesting than broad findings that reinforce the already-known consensus that textual data can reflect systemic gender biases. I suggest that the author lean less towards results and approaches that aggregate findings over the entire dataset. One of the strengths of this work is that it spans so many geographic regions, and I would like to see the findings emphasize this strength more. For example, how much of the variation we see in different measurements of gender bias is due to country of origin rather than subject matter of the textbooks, and vice versa?*

This point is very well taken - we have tried to place greater emphasis on regional variation in findings - for instance changing figures the original Figs 4, 7, and 8, to include a breakdown by region. We have also added the suggested quantitative measure - that "The share of overall variation explained by country and subject is around 8 percent each."

*Some more minor questions and notes:*

*What does it mean for textbooks to be “donor-funded”?*

These are books funded by foreign donors / bilateral development agencies. We have added the following explanation to the paper on page 8: "We also code whether books are funded by a foreign development agency. We include all countries in which textbooks include an acknowledgment of funding by a donor agency. This variable will include some measurement error if funding provided is not acknowledged."

*Section 2.1: “After selecting only files that were in English”; how did you do this?*

This was conducted manually, at the same time as coding the subject and grade of textbooks in the corpus. We have edited the text as follows: “We manually reviewed the materials and selected only files that were in English and that could be classified as learning materials”

*Throughout the paper, some quotation marks are backwards, such as the starting quote around ‘names’ on the end of page 27. If this paper is typeset in LaTeX, the authors should consider using ` as starting quotes instead of ‘.*

Thank you for catching these errors, we have amended them.

*Does the released data that will accompany this paper include the OCR’d textbooks, urls of each source’s landing webpage, or downloaded pdfs’ source links? This could support the use of this dataset by future researchers.*

The released data does include the OCR’d textbooks. It does not include the source URLs - the high rate of "link rot" may make these less useful for future researchers in any case.

*The title of this article could be specified a bit more, e.g. ‘sexist textbooks’ is a bit vague and the title could highlight more of what makes this paper differ from the variety of other research that has quantified the existence of sexism in textual data.*

We have extended the title to add more specificity about the paper, as follows: "Sexist textbooks: Automated analysis of gender bias in 1,255 books from 34 countries"

*Finally, much of the writing in this paper could be improved, and it could benefit from additional rounds of proofreading. For example, “New Analysis of image data” should be “New analyses of image data” on page 28 In addition, page 2 initially specifies 34 countries, but then this switches to 33 in the next paragraph.*

Thank you for catching these errors, we have amended them and carried out a careful copyedit of the paper.

**Reviewer #2:**

*"The paper fulfills the requirements outlined by PLOS ONE: this represents original research and presents results not published elsewhere. The analyses are performed to a rigorous technical standard and are described in sufficient detail. The manuscript is presented in an intelligible fashion and is written in standard English. I appreciate the focus on books from a global sample so that we can further understand general patterns but also who might be exemplars in terms of gender representation. The authors have been very thoughtful in their analyses. This study presents a nice addition to the literature.*

*1. The description of the methods are clear and accessible to a layperson. This is a very exciting toolkit. The authors have taken great care in ensuring that readers can understand the work.*

*2. For the gender representation analysis, it would be interesting to see what the patterns are when including names for which they can identify gender. This could be analysis that goes in an appendix with the caveats of the precision and recall rates.*

We have added the following as a robustness check on line 379: “All of this analysis is conducted with gendered nouns and pronouns and not with gendered names, due to the imprecision in the algorithm correctly identifying names correctly in context. However the results are largely similar if we do include names - with the number of male words increasing to 232,502 and female words to 128,970. Representation by country only changes marginally (see S1 Fig).”

*Similarly, the occupation analysis is likely understating gender skew because of the exclusion of words such as salesman and actress.*

Thank you for pointing this out. We have added this point in the text on line 212 as follows: “For analysis of job type, we discard occupations which occur fewer than ten times in our full textbook corpus, and conservatively discard explicitly gendered

occupations such as sales*man* or actress, which means that if anything we may

understate the gender skew in occupations.”

*3. The authors group books by subject into 14 groups, including “French” distinct from “Language” but not English (though in Figure 2, French is not separated). It is unclear how these groupings were made and why they are separated this way, especially across different countries. If the books are all written in the English language, how can other language books be included?*

Thank you for catching this error. French is mistakenly separated from language in the text on page 12, section 3.1. In practice in our analysis, French is included in the Language grouping, as is shown in Table 2 and Figure 2. Books with the subject French are English-language books for teaching French as an additional language. We have edited the relevant sentence in the text of section 3.1.

*4. For the analysis using the valence, arousal, dominance lexicon (Table 3), would it make sense to have a similar analysis as to what was done in Table 4 in addition to showing the gender parity controls without log GDP?*

We tried running this analysis but did not see much correlation between gender differences in the VAD lexicon and country-level gender parity variables. As a potentially more interesting alternative to showing the variation in gender differences in the VAD lexicon, we generated a new S5 Fig showing average country-level differences.

*5. How should we think about the generalizability of these results? The analysis draws on a broad set of contexts, but they all use English language. How do places that use English medium in their textbooks differ from places that use other languages?*

We have added the following sentences on page 4: "The countries in our sample, in which schooling is at least in part conducted in English, are of course different in one important way to other countries - many having a British colonial legacy. They are not necessarily so different in all relevant dimensions though. The countries cover a range of income levels and geographic regions, and have similar average levels of gender equality to other countries (see S1 Table)." We have also added the relevant Table (S1) to the supplementary information showing how the countries in our sample differ to the rest of the world on several measures of gender equality.

*6. A minor note for the writing: avoid using contractions such as “don’t” and “aren’t”*

Thank you for catching this, we have carried out a careful copyedit of the paper.
