# Supplementary material for: Sexist textbooks: Automated analysis of gender bias in 1,255 books from 34 countries
Source: PLoS One. 2024 Oct 9;19(10):e0310366. doi: 10.1371/journal.pone.0310366 (PMC11463758; doi:10.1371/journal.pone.0310366)
Supplement: Attachment — Submitted filename: Response to Reviewers (round 2).docx [file pone.0310366.s026.docx]

Dear Dr. Kehler,

Thank you very much for your comments and the opportunity to resubmit our paper, we’re delighted for it to be progressing to the next stage.

Below we provide indented responses below each comment from yourself and the two reviewers, which we *reproduce in italics*.

Warm regards,

Lee Crawfurd, Christelle Saintis-Miller, Rory Todd

—-

*Editor:*

*Both reviewers have provided brief comments for your consideration. I’ll add that I agree with Reviewer 1 regarding their comment about dependency parsing not being an alternative to word embeddings. Further, I thought the discussion at the top of page 10 could use some elaboration; for instance, by clarifying that the ‘relationships between’ words are structural, unlike word embeddings. Perhaps a little more detail could be added about what dependency parsing is, and how it differs from the other two methods in the type of information it recovers.*

Thank you for helping us clarify our meaning here - we have removed the statement that dependency parsing is an alternative to word embeddings, and rewritten the introduction to dependency parsing as follows:

“Dependency parsing is a method of syntactic analysis that determines the grammatical structure of sentences by identifying the relationships between words. Unlike word embeddings, which capture semantic relationships based on word co-occurrence patterns, dependency parsing focuses on recovering structural relationships within sentences. Specifically, it uses pre-trained models to infer the grammatical connections between words, providing insights into sentence structure and syntactic roles.

The `universal dependencies' framework \cite{de_marneffe_universal_2021} underpins this approach by first performing 'part-of-speech tagging' - allocating all words in a language to one of a set of syntactic categories (such as adjective, noun, verb, etc.). This categorization depends both on the word itself and the context in which it is used. For instance, in English, `butter' might be categorized as a noun or as a verb (as in `to butter the bread'), depending on its usage in the sentence.”

*Also, as a very minor comment, I noticed that the plural used for ‘corpus’ is ‘corpuses’ on line 460 and ‘corpora’ on line 569.*

Thank you for spotting this inconsistency, we have switched the plural to ‘corpora’ for both cases.

*Reviewer #1: This paper has improved from its original version; thank you.

One main comment for the authors to reflect on further: is it necessary to binarize how you define gender throughout the paper? For example, your name gendering process and exclude names that are ambiguously gendered. I suggest engaging with literature on how gender can be operationalized via text, and the limitations and caveats of how this operationalization could be done (e.g. Cao & Daumé “Toward Gender-Inclusive Coreference Resolution”). “Gender” can be expressed and perceived in a variety of ways, and different operationalizations of gender thus measure different types of gender. Precisely outlining your intentions and definitions of these social constructs is important in bias-related text-as-data work. Otherwise, one risks perpetuating other biases (e.g. exclusion of people whose names may not conform with some types of gender, such as their self-identified gender, or erasure of binary people). Similarly, for age, why must age be analyzed as a binary rather than on the continuum in which it naturally exists? I understand that for some reporting of results (e.g. word co-occurrence), binarizing measurements of social identity can simplify the reporting of results. However, for some findings, such as those related to representation, one could present distributions of names’ gender connotations.*

Thank you for this important reflection on the binary view of gender that is represented in our paper. We have included the following statement as part of our limitations on page 16:

“Fifth, while we recognize that gender can be expressed and perceived in a variety of ways, in this analysis we use a binary view of gender. Part of our reasoning for this more narrow view lies in the books included in our corpus, which only include binary gendered characters. We therefore use the same view in our analysis, though the absence of non-binary characters and adoption of a binary view of gender does reveal something about the norms around gender that are perpetuated by the countries included in our analysis.”

In response to treating age as a continuum, we measure the ‘age’ of characters in the textbooks based on the general meaning of terms included in this analysis (like ‘man’ vs ‘boy’ or ‘grandmother’ vs ‘grandson’). While we recognize that age naturally exists on a continuum, as you point out, we are bound by the terms that occur in the textbooks themselves. Therefore, while we recognize it may be a more crude measure, we feel it is a necessary one in this case.

*With regards to discarding “salesman” and “actress” and possibly misestimating gender skew of occupations, one way of going about this would be to replace all instances of explicitly gendered occupations with a special token or gender-neutral form before conducting textual analyses. For example, replace all instances of “salesman”, “salesperson”, and “saleswoman” with “salesperson” in texts and then compute the association of gender with the token “salesperson” representing one occupation.*

Thank you for this suggestion - we have adopted this proposed approach, and therefore revised Figures 8, 9, S9, S10, S11, S12, and S13. We have edited the text in our methods section as follows: “*We count cases of implicitly gendered occupation terms (such as 'salesman') as equivalent to a co-occurrence of a gendered term and a gender-neutral version of the term ('man' and 'salesperson').*” Results are overall little changed.

*Line 328: “An alternative approach to word embeddings is dependency parsing”. These two methods are not alternatives to each other; they each measure very different things and should yield distinct results. I suggest revising the wording here.*

We have revised this wording as discussed above. *Thank you for providing your data as well!*

*Reviewer #2: Thank you for addressing my comments. My only remaining comment is that you note that you "conservatively discard explicitly gendered occupations such as salesman or actress, which means that if anything we may understate the gender skew in occupations." I suggest adding an appendix exhibit that includes any gendered term (including salesman and actress) to highlight one of the bounds on the gender representation. These contribute to gender inclusion/exclusion.*

As discussed above, we have adopted the suggestion of Reviewer 1 to instead *include* explicitly gendered occupations as equivalent to a co-occurrence of a gendered term and a gender-neutral version of the term.
